# Effects of the Concomitant Activation of ON and OFF Retinal Ganglion Cells on the Visual Thalamus: Evidence for an Enhanced Recruitment of GABAergic Cells

**DOI:** 10.3389/fncir.2015.00077

**Published:** 2015-11-24

**Authors:** Giovanni Montesano, Marcello Belfiore, Maddalena Ripamonti, Alessandro Arena, Jacopo Lamanna, Mattia Ferro, Vincenzo Zimarino, Alessandro Ambrosi, Antonio Malgaroli

**Affiliations:** ^1^Neurobiology of Learning Unit, Division of Neuroscience, Scientific Institute San RaffaeleMilan, Italy; ^2^Università Vita-Salute San RaffaeleMilan, Italy; ^3^Ophthalmology, Azienda Ospedaliera San PaoloMilan, Italy

**Keywords:** dLGN, retina, 4-AP, 4-aminopyridine, inhibitory neurons, visual thalamus, c-Fos, NeuN

## Abstract

A fundamental question in vision neuroscience is how parallel processing of Retinal Ganglion Cell (RGC) signals is integrated at the level of the visual thalamus. It is well-known that parallel ON-OFF pathways generate output signals from the retina that are conveyed to the dorsal lateral geniculate nucleus (dLGN). However, it is unclear how these signals distribute onto thalamic cells and how these two pathways interact. Here, by electrophysiological recordings and c-Fos expression analysis, we characterized the effects of pharmacological manipulations of the retinal circuit aimed at inducing either a selective activation of a single pathway, OFF RGCs [intravitreal L-(+)-2-Amino-4-phosphonobutyric, L-AP4] or an unregulated activity of all classes of RGCs (intravitreal 4-Aminopyridine, 4-AP). In *in vivo* experiments, the analysis of c-Fos expression in the dLGN showed that these two manipulations recruited active cells from the same area, the lateral edge of the dLGN. Despite this similarity, the unregulated co-activation of both ON and OFF pathways by 4-AP yielded a much stronger recruitment of GABAergic interneurons in the dLGN when compared to L-AP4 pure OFF activation. The increased activation of an inhibitory thalamic network by a high level of unregulated discharge of ON and OFF RGCs might suggest that cross-inhibitory pathways between opposing visual channels are presumably replicated at multiple levels in the visual pathway, thus increasing the filtering ability for non-informative or noisy visual signals.

## Introduction

The lateral geniculate nucleus (LGN), which allows information transfer from the retina to the visual cortex, has a complex role in vision that is still not fully understood and clearly must go beyond the simple regulation of transfer efficiency of visual signals (Hubel and Wiesel, 1961; Guillery and Sherman, 2002). This structure is composed of a variety of intrinsic neuronal cells that can be categorized into long range relay neurons connecting to the visual cortex, the thalamocortical neurons, and GABAergic interneurons which are randomly scattered within the LGN (Gabbott and Bacon, 1994). Both cell types are targeted by the input from retinal ganglion cells (RGCs), known to invade the LGN from its lateral border (Kim et al., 2010; Dhande et al., 2011), forming large rosette-like presynaptic boutons on thalamic cells (Li et al., 2003). The activation of interneurons by the retino-thalamic axons provides feedforward inhibition of thalamocortical neurons. This inhibitory input controls the number and the precision of visually evoked spikes and refines the receptive fields of thalamocortical neurons (Berardi and Morrone, 1984; Blitz and Regehr, 2003, 2005). Thalamic neurons are also reached by a very large number of non-retinal inputs which originate from the thalamic reticular nucleus, the superior colliculus, layer 6 cortical neurons [i.e., the cortico-thalamic feedback, which enters the dLGN from its medial border (Jacobs et al., 2007; Su et al., 2011)], the pedunculopontine tegmentum, the parabigeminal nucleus, and the pretectum (Bickford et al., 2000). In other mammals, evidence exists that this nucleus might also be reached by afferents from the substantia reticularis (Francesconi et al., 1988). The synaptic contacts made by non-retinal inputs, together with those of *in situ* GABAergic interneurons, account for the large majority of LGN synaptic connections (Van Horn et al., 2000). They participate in visual perception and its modulation, for example during the different sleep-wake states.

In rodents, the LGN complex is usually subdivided in its dorsal portion (dLGN), the intergeniculate leaflet (IGL; the pregeniculate nucleus of primates), and in the ventral lateral geniculate nucleus (vLGN). While the IGL and the vLGN belong to the circadian rhythm system (Morin and Allen, 2006), the dLGN is the image forming region of the LGN. Based on conventional histological stainings, the LGN lamination seen in high mammals (Polyak, 1957; Hubel and Wiesel, 1961) is not visible in the rodent dLGN. In fact, most of RGC axons cross at the optic chiasm to reach the dLGN of the opposite brain hemisphere. Ipsilateral retinal axons which represent just the ~3–5% of the total RGCs fibers (Polyak, 1957; Jeffery, 1984) segregate in a specific dLGN subregion (Godement et al., 1980, 1984; Reese, 1988), a rostro-ventral central structure named the “hidden lamina,” suggestive of a primordial lamination plan (Reese, 1984, 1988). This finding was confirmed and extended by more refined labeling methods based on transgenic lines expressing fluorescent proteins in specific subsets of RGCs (Huberman et al., 2008, 2009; Kim et al., 2010; Rivlin-Etzion et al., 2011). These anatomical findings need to be substantiated by functional recordings to evaluate the relevance of these neuronal-synaptic laminae. Interestingly, electrophysiological recordings from rat LGN have demonstrated a comparable response to ipsilateral and contralateral stimuli on a large fraction of dLGN neurons, suggesting a much larger superimposition of crossed and uncrossed axon collaterals (Grieve, 2005). Based on these results binocular integration in rodents might already occur inside the visual thalamus (Grieve, 2005). The functional organization of the dLGN activation pattern can be established by the expression of the c-Fos gene product, a member of the Immediate Early Gene family, whose expression is calcium regulated through CREB (cAMP response element-binding protein) phosphorylation (Sheng et al., 1990; Flavell and Greenberg, 2008). In fact, cellular levels of the c-Fos protein have been shown to be able to report neuronal firing and synaptic regulation (Murphy et al., 1991). Based on immunocytochemical detection or using specific mouse lines where the c-Fos promoter drives the expression of reporter molecules (Greferath et al., 2004; Murphy et al., 2004), staining for c-Fos can be effectively used to trace the spatial distribution of active cells such as those of the visual thalamus (Montero and Jian, 1995; Correa-Lacarcel et al., 2000; Greferath et al., 2004; Lu et al., 2004; Dai et al., 2009). In most of these studies, the light stimulation induced the appearance of some c-Fos positive cells in the dLGN, although no functional segregation or lamination of active cells was reported (Montero and Jian, 1995; Correa-Lacarcel et al., 2000; Greferath et al., 2004).

The aim of our study was to characterize the effects of two different (chaotic or highly coherent) retinal inputs to the dLGN in terms of different patterns of c-Fos expression in this nucleus. We used monocular ON-OFF light pattern stimulation in association with exposure of RGCs to either 4-Aminopyridine (4-AP) or L-(+)-2-Amino-4-phosphonobutyric (L-AP4). 4-AP was used to produce random increase in the excitability of both ON and OFF ganglion cells, hence in their unregulated firing. On the contrary, L-AP4, an ON-pathway inhibitor (Slaughter and Miller, 1981), was applied to promote a much more coherent RGC firing. Our results provide evidence that these two modalities of retina activation, which are expected to convey different amounts of visual information, based on their low or high level of coherence in RGC activities, both activate the intrinsic inhibitory circuit of the dLGN but to a different extent. In the presence of noisy or non-coherent input, as during 4-AP administration, this inhibitory network of dLGN GABAergic interneurons appeared to be more strongly recruited, possibly in a physiological attempt aimed at filtering out non-coherent or noisy signals. Interestingly, the vast majority of active neurons appeared not randomly distributed across the dLGN, with a higher density of active cells near the lateral border. Our findings provide insights into the functional architecture of the rat dLGN and suggest that the activity pattern of dLGN cells follows to some extent but do not superimpose with the well-known distribution of crossed retino-geniculate fibers (Huberman et al., 2008, 2009; Kim et al., 2010; Rivlin-Etzion et al., 2011).

## Materials and methods

### Animal care procedures

Research and animal care procedures were approved by our Institutional Animal Care and Use Committee for Good Animal Experimentation in accordance with the Italian Ministero della Salute code of practice for the care and use of animals for scientific purposes (IACUC number: 54o). Experiments were carried out on adult male Sprague Dawley rats (weight 175–200 g). Before experiments, animals were individually caged with free access to food and water *ad libitum* and were exposed to 12 h light/dark cycles at 23°C constant room temperature. For the c-Fos expression experiments, to obtain a pure monocular light response, the functionality of right eye was abolished. This procedure was applied because, although the input of ipsilateral retinal axons is just ~3–5% of the total fiber input (Polyak, 1957; Jeffery, 1984), functional recordings have demonstrated a large dLGN responses to ipsilateral stimuli suggestive of a large divergence of uncrossed axons (Grieve, 2005). The alternative procedure, i.e., suturing of the eyelid above the eyeball with or without the application of dark glue material on its external surface, was not selected because of potential tonic and non-coherent activation of the retina by light filtering through the eyelid and/or by increased external pressure on the eyeball. To this aim, animals were deeply anesthetized with sevoflurane (Sevorane, Abbott) and their retina surgically removed. After surgery, we applied a local anesthetic (Lidocaine) to reduce animal discomfort. An antibiotic powder (Cicatrene, Johnson and Johnson) was then added followed by the right eyelid suturing to prevent infections. Rats were then housed in darkness for 48 h before experimenting to reduce background levels of c-Fos expression arising from ambient light and from the right eye surgical procedure. In these conditions no change in the activity of the dLGN contralateral to the removed eye could be detected when comparing the number of c-Fos positive cells between the two dLGNs (ispilateral and contralateral to the enucleated eye) of rats kept in darkness for 2 h when one eye was enucleated (*p* > 0.10). Surgery was always performed under sevoflurane anesthesia and animals were sacrificed with an overdose of sodium pentobarbital.

### Ganglion cell recordings from isolated retinas

Retinal preparations were obtained as described in a published protocol (Schmidt and Kofuji, 2011). Briefly, rats were dark adapted for several hours and then euthanized in a dark ambient via CO_2_ asphyxiation. Always minimizing light exposure, both eyes were enucleated and opened by puncturing the eyeball at the limbus. Then the cornea was removed with a circular incision and the lens removed with forceps. Finally, forceps were used to tear up the sclera freeing the retina completely. The retina was then cut in four pieces. To avoid long-term changes by pharmacological manipulations, only one recording was obtained from each piece of the retina, which was then discarded. Every step after the removal of the sclera were performed in CO2/O2 saturated Ames' medium (Sigma) with 0.19% Sodium Bicarbonate. The same solution was used to maintain the tissue after dissection. Retina preparations were allowed to dark adapted for 1 h prior to use. Loose patch recording from single RGCs were obtained from these acute retina preparations of rat retinas using 5–8 MOhm electrodes filled with Ames' medium. During electrophysiological recordings, the quartered pieces of the retina were continuously superfused with CO_2_/O_2_ saturated Ames' medium kepts at room temperature (24°C). Electrophysiological Signals were acquired using an Axopatch 200B amplifier (Axon Instruments, Foster City, CA), filtered at 2–5 kHz and digitally acquired at 20 kHz using a 16-bit analog-to-digital interface (Digidata, Axon) controlled by the pClamp acquisition software. During recordings, drugs (4-Aminopyridine, 4-AP, 0.02 mM) were dissolved in Ames' medium and applied through the bath perfusion system. If not otherwise indicated, salts and chemicals were obtained from Sigma-Aldrich (Sigma-Aldrich, St. Louis, MO). Light stimuli consisted of full field illumination of the retinal preparation using a LED placed under the microscope condenser. The light pulses lasted 1 s with 19 s interval between pulses. Thirty sweeps were recorded before during the application of 4-AP for every analyzed cell. In the described conditions retinas were available for recording for up to 8 h after dissection. In preliminary experiments we could obtain stable recordings from single cells for 1.5 h.

As opposed to 4-AP, L-AP4 is a widely known tool for investigating the retinal circuitry and its effects on such circuit have been extensively studied since 1981 (Slaughter and Miller, 1981). We were able to reproduce such known effects in our experimental setting but no formal analysis was performed (see Supplementary Figure 1 for an exemplar recording).

### Visual stimulation

For light stimulation, we placed animals inside a Perspex tube leaving out their neck and head. The head was clamped by a custom device, which lowered the head to an angle of 30° with respect to the animal horizontal plane. Animals were then positioned in front of a cathode flat monitor (100 Hz refresh frequency). To induce the activation of ON-OFF retinal fields, rats were visually stimulated with black and white vertical bars [height = 30 cm, width = 2 cm, irradiance: white bars 37 mW/m^2^; black bars 0.11 mW/m^2^ at a wavelength of 509 nm, the peak of rat M-cones absorbance (Jacobs et al., 2001)]. By angling the screen to 45° (with respect to the animal longitudinal rostro-caudal axis) the monitor was placed in front of the animal left eye. The distance from the screen was set to obtain a spatial frequency of 0.126 cycles/degree. In the experiments presented here, we used a pattern reversal at 2 Hz and the stimulation protocol lasted for 2 h. Based on a set of preliminary experiments, this length of the experiment was set to allow optimal c-Fos protein expression levels. The day of the experiment, we kept the no-light group in the dark for 2 h in the same experimental setting as in the light stimulation experiments except for the fact that the monitor was turned off. To determine if light activation protocols and other manipulations (intravitreal drug administrations) were indeed effective, the c-Fos activation pattern of RGCs was first analyzed. When light induced c-Fos enhancement could not be detected in the retina, the brains of the corresponding animals were discarded and not further processed. Only two rats were excluded based on this criterion and in both cases the retinal stimulation was not effective due the presence of blood filling the vitreal chamber after drug injection.

### Intravitreal drug administration

For drug treated subgroups, animals were injected intravitreally either with the selected drug or with physiological saline and then kept in the darkness for 30 min before experimenting to allow drug equilibration. For the injections, rats were deeply anesthetized with sevoflurane and injected intravitreally with the selected drug using a 10 μl Hamilton syringe coupled to the 30G eye injection needle through a Teflon tubing (TE50) filled with mineral oil. The injection site was in between the cornea and the sclera (limbus), and the appropriate intravitreal needle position was determined by visual inspection through the iris. The injection volume was set to 5 μl. L-(+)-2-Amino-4-phosphonobutyric (L-AP4, Tocris Bioscience) and 4-aminopyridine (4-AP, Sigma Aldrich) were diluted in a Tyrode saline solution containing: NaCl (119 mM), KCl (5 mM), Hepes (25 mM) CaCl2 (2 mM), MgCl2 (2 mM), Glucose (6 g/L), which was buffered to pH 7.4. The final concentration for both drugs in the vitreous chamber was approximately 2 mM. Right eyes were injected for Visual Evoked Potential (VEP) experiments and left eyes for c-Fos experiments.

### Immunolabelings

At the end of the stimulation protocols, rats were sacrificed with an overdose of sodium pentobarbital (Thiopental, intraperitoneal injection 30 mg/Kg; Inresa-LD) within a time span from 02:00 p.m. to 06:00 p.m. Via the ascending aorta, we quickly perfused the circulation by gravity, first with a cold buffered solution containing heparin (0.12 M phosphate buffer, 4°C; Heparin 5000 UI/ml) followed by 4% paraformaldehyde dissolved in 0.12 M sodium phosphate buffer. At the end of the perfusion, brains were removed and post-fixed overnight in the same fixative solution (4°C). Brains were included in 4% agar and coronal sections (35 μm thick) were obtained at regular space intervals to reconstruct the lateral geniculate nucleus (LGN) from both hemispheres slices using a Vibratome (Leica VT1000 S Vibrating Blade Microtome). To extract the retina, after intra-cardiac perfusion with fixative, the eye was removed and the cornea gently sectioned. The eye-cup was then post-fixed in 4% paraformaldehyde solution overnight (4°C). The whole retina was gently removed and left free floating in a multi-well dish. For immunolabeling, free-floating sections and retinas were initially washed with a solution containing: 0.12 M phosphate buffer, 0.3% Triton X-100, 1% BSA, pH 7.4. Slices and retinas were incubated overnight at 4°C with a mixture of primary antibodies dissolved in the same solution (rabbit anti c-Fos, 1:300 dilution, Santa Cruz Biotechnology K-25; IgG1 mouse anti NeuN, 1:300 dilution, Millipore, MAB377; IgG2A mouse anti GAD65 and anti GAD67, 1:300 dilution; CHEMICON MAB351 and MAB5406). After incubation with primary antibodies, retinas and slices were extensively washed and incubated for 2 h at room temperature with a mixture of species-specific fluorescent secondary antibodies (anti rabbit-CY5 or anti rabbit-Alexa647 conjugated donkey anti mouse IgG, 1:200 dilution; anti mouse-FITC conjugated donkey IgG, 1:200 dilution; Jackson ImmunoResearch; Southern Biotech, A-21126, anti mouse IgG2A, FITC conjugated donkey, 1:200 dilution; Molecular Probes, 1080-02, anti mouse IgG1, Alexa647 conjugated donkey, 1:200 dilution). After extensive washing, brain sections and retinas were mounted on glass slides and covered with coverslips using an aqueous mounting medium (Fluorsave, MERK). In a set of preliminary experiments, we tested for the specificity of primary and secondary antibodies and we excluded cross-reactivity. In addition, we evaluated the level and the excitation-emission spectrum of background fluorescence of retinas and of the LGN structure. For the detection of c-Fos expression, we used secondary antibodies emitting in the far-red (CY5 or Alexa647 fluorescent probes) since in this portion of the spectrum the auto-fluorescence of the LGN tissue is very faint.

### Image acquisition and analysis

Fluorescent images were acquired for every slice (400 μm) by one photon confocal microscopy (10× –63× magnification; Axioscope LSM 510, Zeiss) and fused in composite images or collages to reconstruct every LGN section. Based on a set of preliminary experiments we determined the best acquisition parameters (excitation intensity, gain, and pinhole settings; no background subtraction) used throughout this study. For each animal, the LGN analysis was restricted to *n* = 9 sequential and equally spaced coronal brain slices (including both hemispheres). Composite images of ipsilateral and contralateral hemispheres were band-pass filtered with a Fast Fourier Transform algorithm, fluorescent cells detected by a particle analyser tool and segmented using the Watershed algorithm (ImageJ, NIH). The best threshold for detection was established for every filtered image according to the distributions of the fluorescent signal and background noise (Max Entropy algorithm, ImageJ, NIH). In addition, all detected NeuN and c-Fos positive cells were visually scrutinized before final counting. Although the analogic level of c-Fos cell activation provides important information, we preferred to concentrate on a more elementary digital classification (number of active vs. inactive cells) because: (i) the time frame to combine a thorough spatial analysis with accurate estimates of cell-fluorescence (requiring high magnification and specific acquisition settings) was disproportionate large; (ii) c-Fos intensities showed unwanted slice to slice variability and also more subtle local changes because of heterogeneity in background fluorescence and/or in the efficacy of the staining procedure; (iii) depending on the timeframe and degree of activation the c-Fos protein doesn't remain concentrated in the nuclear and perinuclear areas but diffuses away in dendritic branches, often out of focus. To attribute cells to one of the three LGN subdivisions (dLGN, vLGN, and IGL), it was critical to define the borders of these structures more precisely. The boundaries derived from the detectable anatomical features and from the characteristic spatial distribution of NeuN positive cells in these nuclei. For counting of c-Fos/NeuN single and double positive cells, neurons were detected either in the NeuN channel or in the c-Fos channels. Based on their morphology and fluorescence values (see above), neurons were considered single or double positive NeuN/c-Fos cells. This analysis and the characterization of their spatial distribution was obtained by custom routines written in Matlab (Mathworks). A Matlab routine was developed to subdivide every dLGN coronal section in a series of stripes of equal thickness, running parallel to the lateral edge of the dLGN. C-Fos and NeuN positive cells were counted in every stripe, values refer to crude counts or counts normalized for stripe area. The thickness of these stripes was selected to avoid disproportionate fluctuations of the cell-count index essentially when the number of cells/stripe drops to a very low value. For every dLGN, after searching for the section with highest count of cells labeled by NeuN, the stripe thickness was then set in all sections according to the square root of this value.

### Visual evoked potentials (VEPs) recordings

For VEP recordings, we used Sprague-Dawley rats matched for strain, age, and sex with those used in c-Fos experiments (weight 175–200 g). Very small stainless steel screws (diameter ~1 mm) were positioned above the left and right binocular visual cortex and used as recording electrodes. For electrode positioning, rats were deeply anesthetized with the volatile anesthetic sevoflurane (Sevorane, Abbott), the skull was exposed and using a stereotaxic apparatus to find the correct stereotaxic coordinates of the binocular visual cortex, small holes were drilled in the skull without exceeding the bone thickness. The electrodes were screwed and connected to gold pin terminals, a liquid methacrylate resin (REPORT/N Salmoiraghi Produzione Dentaria Srl, Italy) was polymerized above them leaving just the electrode endings exposed. After surgery, gentamicin-sulfate (1.5 mg/Kg; i.p. injection twice a day) and dexamethasone-phosphate (0.2 mg/Kg; one subcutaneous injection) were administered. Three days after surgery, animals were prepared for experiments. Rats were deeply anesthetized and a sterile polyethylene catheter was positioned inside the trachea for mechanical ventilation (SERVO ventilator 900C—Siemens, 3% Sevoflurane). A volume-controlled ventilator delivered a tidal volume of 6 ml with a respiratory rate of 80–85 breath cycles/min and volume of 0.5 L/min. A gas analyser (Vamos, Dröger) monitored the inspiratory and the expiratory concentrations of the anesthetic and CO_2_ level. Rats were placed on an anti-vibration breadboard over a heating pad to maintain their body temperature in the physiological range (36–37°C). In order to obtain muscle relaxation, rats were curarized (Atracurium, 5 mg/kg, caudal vein injection every 20 min). During experiments, rats were kept in a dark environment, the left eye was covered by a black patch and the right eye stimulated with a white LED [trains of 300 ms light pulses, 0.1 Hz, *n* = 30 per train; white LED irradiance was set to 111 μW/cm^2^ measured at the 509 nm wavelength, the peak of rat M-cones absorbance Jacobs et al., 2001]. Gold-pin electrodes were connected to a custom-made DC-decoupled amplifier with a fixed gain (G_IN_ = 100). The differential signals coming from the amplifier were low-pass filtered (GPRE = 50, *f*_*p*_ = 1 KHz) and further amplified (210 A amplifier, Brown Lee Precision). Data were digitized at 20 KHz sampling frequency using a ITC18 16-bit data acquisition interface (HEKA), connected to a computer running a custom data acquisition software developed in LabVIEW environment (National Instruments). For VEP measurements, a differential recording was obtained between the left and the right V1 skull electrodes. The ground potential was obtained by an additional hook electrode positioned on the external ear. The animals together with the first amplification stage, was kept inside a Faraday cage. VEPs were recorded before and after intravitreal drug administration using the same recording and light stimulation protocols.

## Data analysis and statistical methods

### Spike train analysis

Autocorrelograms were calculated on recordings of 2 min of spontaneous activity with a lag window of 2 s (from −2 s to +2 s) with 100 ms bin-size and normalized setting the lag-zero counts equal to one. The power spectra were obtained as the Fourier transform of the autocorrelograms. Periodic and non-periodic behaviors were selected based on an Periodicity Index calculated as the height of the highest peak in the normalized power spectrum divided by the length of its base. The height of the peak was calculated as the difference from the lowest surrounding minima and only peaks above a defined threshold were considered (0.008). If no peaks could be detected or the Periodicity Index was to low (below 0.003), the cell was classified as non-periodic. The selectivity of the cell responses for the light stimuli was calculated as a Discrimination Index = (A1 − A2)/(A1 + A2), where A1 is the number of spikes during the light pulse and A2 is the number of spikes right after the stimulus calculated on a time window of the same duration as the light pulse. It is positive for ON cells and negative for OFF cells and tends to zero if there is an equal probability of firing during and after the stimulus. The entropy of the Interspike Interval (ISIs) was calculated as H $=\underset{\text{i}}{\sum }\left({\text{ISI}}_{\text{i}}∕\underset{\text{j}}{\sum }{\text{ISI}}_{\text{j}}\right)\text{x log}\left({\text{ISI}}_{\text{i}}∕\underset{\text{j}}{\sum }{\text{ISI}}_{\text{j}}\right)$ on a peristimuls time window of 4 s (1 s before and 1 s after the light pulse). The Standardized Entropy (Hs) was then obtained dividing by the maximum entropy value of each ISI series [calculated as log(n) where n is the number of ISIs in the series] to account for differences in the number of spikes. All calculations were made for each cell in each experimental condition on a single trace obtained as the superimposition of 30 sweeps. Finally, the significance of the effect of 4-AP on the discrimination ability of the cell was tested using a logistic regression model with a Logit link function where logit(p) = log(p/(1−p)). The model included random effects to account for clustered observations coming from retinal preparations of the same rat (Rat random effect) and from the same cell (i.e., before and after 4-AP, Cell random effect). In this model, the spikes were counted using the same time window as in the Discrimination Index, but were classified as successes and failures: spikes were considered as successes if they occurred during the light pulse for ON cells or after the light pulse for OFF cells. Otherwise they were counted as failures. Successes and failures were used as the response variable to match the assumption of binomial distribution of the data.

### Monte Carlo methods and spatial analysis

Monte Carlo methods were used to generate random samples of activated cells in the dLGN using the experimental NeuN data sets. To compare the simulated random activated cell patterns to the experimental observations, we measured the entropy of the cell distribution for each analyzed brain LGN section. To calculate the entropy values we used the Voronoi tessellation generated by each cell-point pattern, for both random and experimental data sets. For the Voronoi tessellation all locations were associated in space with their closest member(s) using the Euclidean distance (Okabe et al., 2000). The Voronoi diagram composed by ordinary Voronoi polygons was used to compute the entropy of each point pattern on a finite surface with a slightly modified version of the Shannon's equation for entropy (Chapman, 1970):

$$H_{c-Fos}=-\sum_{i\;=\;1}^{n_{c-Fos}}\frac{A_{i}}{\sum_{j\;=\;1}^{n_{c-Fos}}A_{j}}\cdot \ln\frac{A_{i}}{\sum_{j\;=\;1}^{n_{c-Fos}}A_{j}}$$

where *n*_*c*−*Fos*_ is the total number of the points (cells) generating the observed pattern, *A*_*i*_ is the area of the Voronoi polygon associated to point *i*. For each dLGN section, we computed the entropy *H*_*sim*_ for *N*_*sim*_ = 50.000 random samples drawn from the NeuN set, with sample size *n*_*c*−*Fos*_. For each slice, the *p*-value of the observed entropy value *H*_*c*−*Fos*_ was calculated as *p* = *n*∕*N*_*m*_ where n is the number of simulated entropy values *H*_*sim*_ less than to the observed entropy *H*_*c*−*Fos*_.

To compare neuronal density in brain sections, we used frequency counts in k dLGN stripes (double positive NeuN and c-Fos neurons). To verify the null hypothesis of the uniform distribution across stripes, a *G*-test was performed on the observed frequencies of NeuN cells. To take into account the reduction in the area of stripes due to the dLGN curvature, the expected frequency in each stripe was normalized to the ratio of the stripe area over the whole dLGN section area. Hence the test statistic becomes:

$$G=\text{2}\cdot \sum_{i\;=\;1}^{k}O_{i}\cdot \ln\frac{O_{i}}{E_{i}}$$

Where *k* is the number of stripes the dLGN section was subdivided into, *O*_*i*_ is the observed frequency in each stripe and *E*_*i*_ is the expected frequency in each stripe calculated as:

$$E_{i}=\frac{S_{i}}{\sum_{i\;=\;1}^{k}S_{i}}\cdot n_{NeuN}$$

where *S*_*i*_ is the area of each stripe and *n*_*NeuN*_ is the total number of NeuN cells in the dLGN section. Under the null hypothesis, the test statistic has χ^2^ distribution with *k-1* degrees of freedom. To verify if the median value of double positive NeuN/c-Fos neurons was smaller (closer to the lateral edge) than the median value of the NeuN cell population, for each dLGN section *N*_*sim*_ random samples were extracted from the NeuN set (sample size *n*_*c*−*Fos*_). Then, for each sample the position of each cell along the latero-medial axis was calculated as before and the median value for each sample calculated. The *p*-value was calculated as above.

### VEP analysis

To better visualize coherence of VEPs pattern across successive trials, we plotted the recorded voltage values over time for each trial or sweep in a heat-map graph. In order to evaluate possible changes of the ON and OFF responses induced by L-AP4 and 4-AP treatments, we analyzed individual VEP trials in the 0–150 ms time window (ON response) and in the 300–450 ms time window (OFF response). For each trial we computed the height of the first positive peak in the OFF and ON responses, and the integral of the root mean square or quadratic mean (RMS) for the ON and OFF responses in the two time windows listed above. The presence of significant differences between treatments was evaluated by means of permutation statistical methods based on t-type statistics, to avoid any assumption on parental distribution (Pesarin and Salmaso, 2010). *P* < 0.05 were considered significant. All VEP statistical analyses were performed in R environment.

### Statistical test to analyze significant differences in cell counts

To asses possible differences in cell counts between different treatments, we fitted a Generalized Linear Mixed Model (GLMM) to the data (McCulloch et al., 2008). In more details, the observed number of c-Fos positive cells in each portion of the dLGN was considered as a random variable with Poisson distribution. The parameter λ of the Poisson distribution was allowed to depend both on the area of the considered dLGN structure and the applied treatment with a logarithmic link function.

$$\ln\lambda =(\alpha_{0}+\alpha_{1}{\text{I}}_{\text{t}})+(\alpha_{2}+\alpha_{3}{\text{I}}_{\text{t}})\cdot \text{area}$$

where I_t_ is the dummy variable that identifies the treatments to be compared, *area* is the area of each dLGN area section. In such parameterization, α_0_ identifies the intercept relative to the first treatment, α_1_ is the difference of the intercepts of the two treatments, α_2_ identifies the slope of the first treatment and α_3_ is the difference between the slopes. We added a random effect to take into account possible dependencies among brain slices belonging to the same rat. The null hypothesis, H_0_:α_1_ = α_3_ = 0 was verified by means of the Likelihood Ratio test statistic with p-values computed by means of bootstrap (Faraway, 2006; McCulloch et al., 2008). Statistical analyses were performed in Matlab and R environment.

### Statistical test to analyze significant differences in c-Fos expression of GAD-positive cells

GAD-positive cells were identified on 63× confocal raw images. Then, a circular area of 20 pixel radius centered on each GAD positive cell was used to measure c-Fos expression. For every image, a distribution of the c-Fos background was generated sampling the image with 20 pixel radius circular areas (avoiding previously selected putative c-Fos positive cells) several times (1000) and this was used as the null hypothesis distribution to test the value of c-Fos expression of each GAD positive cell. Fluorescence values greater than the mean of the background plus twice the standard deviation of the background were used to identify c-Fos positive cells among GAD positive interneurons (see Supplementary Figure 2). A logistic regression was then used to test the effect of drug treatment over c-Fos expression in these neurons. A similar method was employed to test the even distribution of GAD positive cells, where a generalized linear model for counting processes was used to assess the homogeneity of cells counts across all locations, and the probability of high c-Fos expression at a given lateral-medial location, again using a logistic regression (see Results).

## Results

### Effects of 4-AP on isolated rat retinas

**Figure 1:**
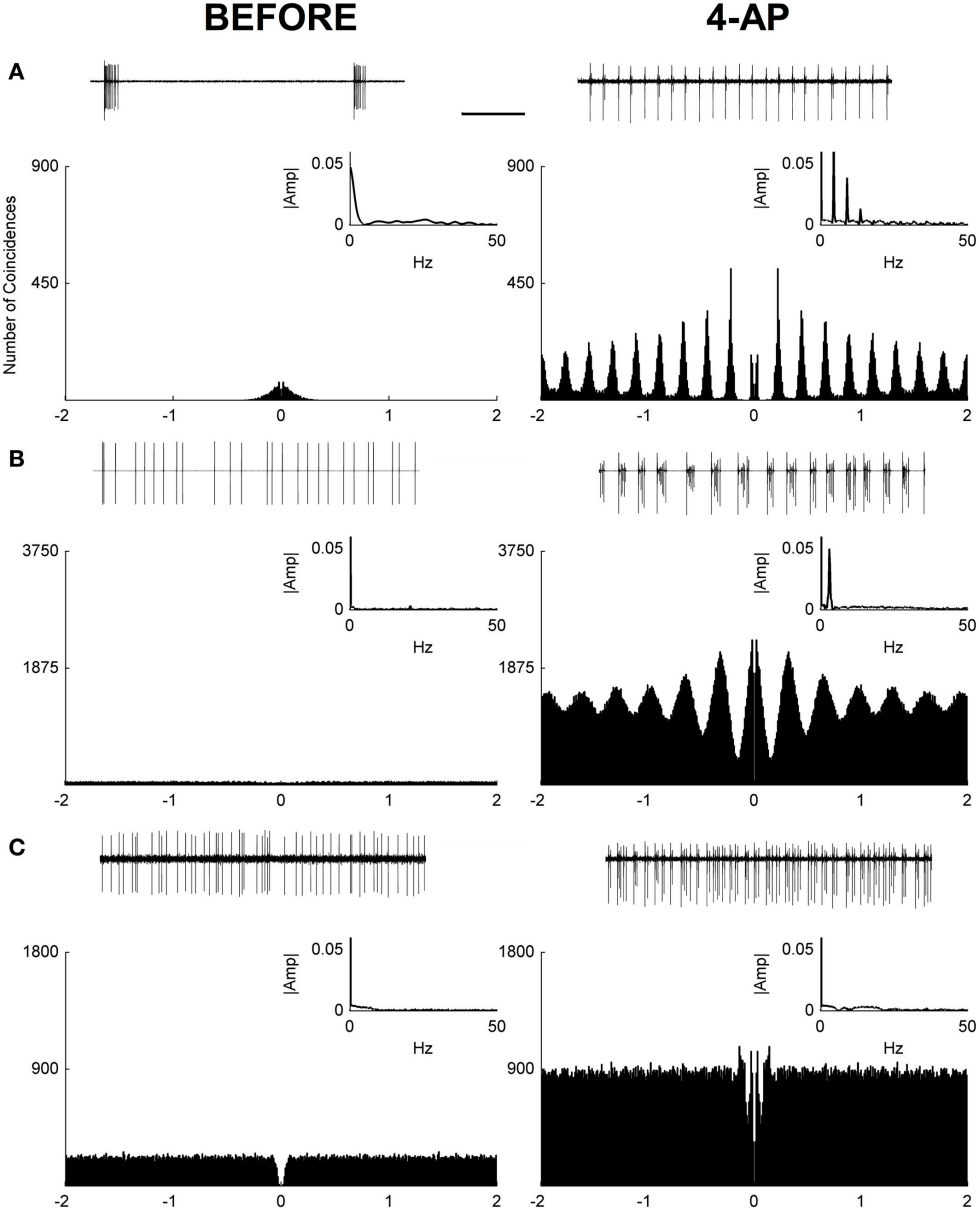
**Autocorrelograms and power spectra of RGC spontaneous activity recorded from rat isolated retinas**. **(A–C)** Spike extracellular recordings (top traces), autocorrelograms (main plot), and power spectra (small insets) in three exemplar cells before (left) and during (right) the application of 4-AP (0.02 mM). In some cases (12 out of 20 RGCs) a periodic behavior was observed in the presence of 4-AP with frequencies in the range of 2.2–5 Hz (mean ± STD = 3.52 ± 0.86). Three different behaviors could be detected in the 4-AP conditions, periodic non-bursting **(A)**, periodic bursting **(B)**, non-periodic **(C)**. The periodic non-bursting behavior was characterized by groups of one or two spikes repeated at regular intervals with sharp peaks in the autocorrelogram and harmonic components in the power spectrum. For periodic bursting, highly regular bursting activity, with dilated peaks in the autocorrelogram and no armonic components in the power spectrum was observed. The non-periodic behavior consisted of a flat autocorrelogram and no clear dominant frequencies in the power spectrum. The black scale bar (1 s, top of panel **A**) applies to all spike recording traces **(A–C)**.

In this study, loose-patch recordings from single RGCs in isolated rat retinas were used to investigate the effects of 4-Aminopyridine (4-AP) on the RGC firing rate. Recordings were obtained from 20 RGCs from 8 animals. The firing rate before and after the application of 4-AP (0.02 mM) was obtained in each RGC recording. In both conditions (before and after 4-AP) spontaneous activity and the evoked response to light stimulation were recorded. In these experiments spontaneous activity was evaluated over a period of 2 min before and after 4-AP, always preceded by a brief period of dark adaptation (5 min). The results showed that after the application of 4-AP the spontaneous activity of ganglion cells was generally increased (mean fold increase = 6.2, IQR = 5.17, see Figure 1). Since in some cells a shift to a periodic firing pattern was detected following the application of 4-AP, autocorrelograms of spontaneous spike series were calculated to evaluate such periodicities. Periodic behavior was assessed via the analysis of Power Spectra and the calculation of a Periodicity Index (see Materials and Methods). Based on this analysis 12 over 20 cells showed periodic firing behavior at low frequencies after the application of 4-AP in the range of 2.2–5 Hz (mean predominant frequency = 3.52 Hz, STD = ± 0.86). Three major classes of cells could be identified: periodic non-bursting, periodic bursting, and non-periodic (Figure 1). The periodic non-bursting behavior was characterized by groups of one or two spikes repeated at regular intervals with sharp peaks in the autocorrelogram and harmonic components in the power spectrum. For periodic bursting, highly regular bursting activity, with dilated peaks in the autocorrelogram and no armonic components in the power spectrum was observed. The non-periodic behavior consisted of a flat autocorrelogram and no clear dominant frequencies in the power spectrum.

**Figure 2:**
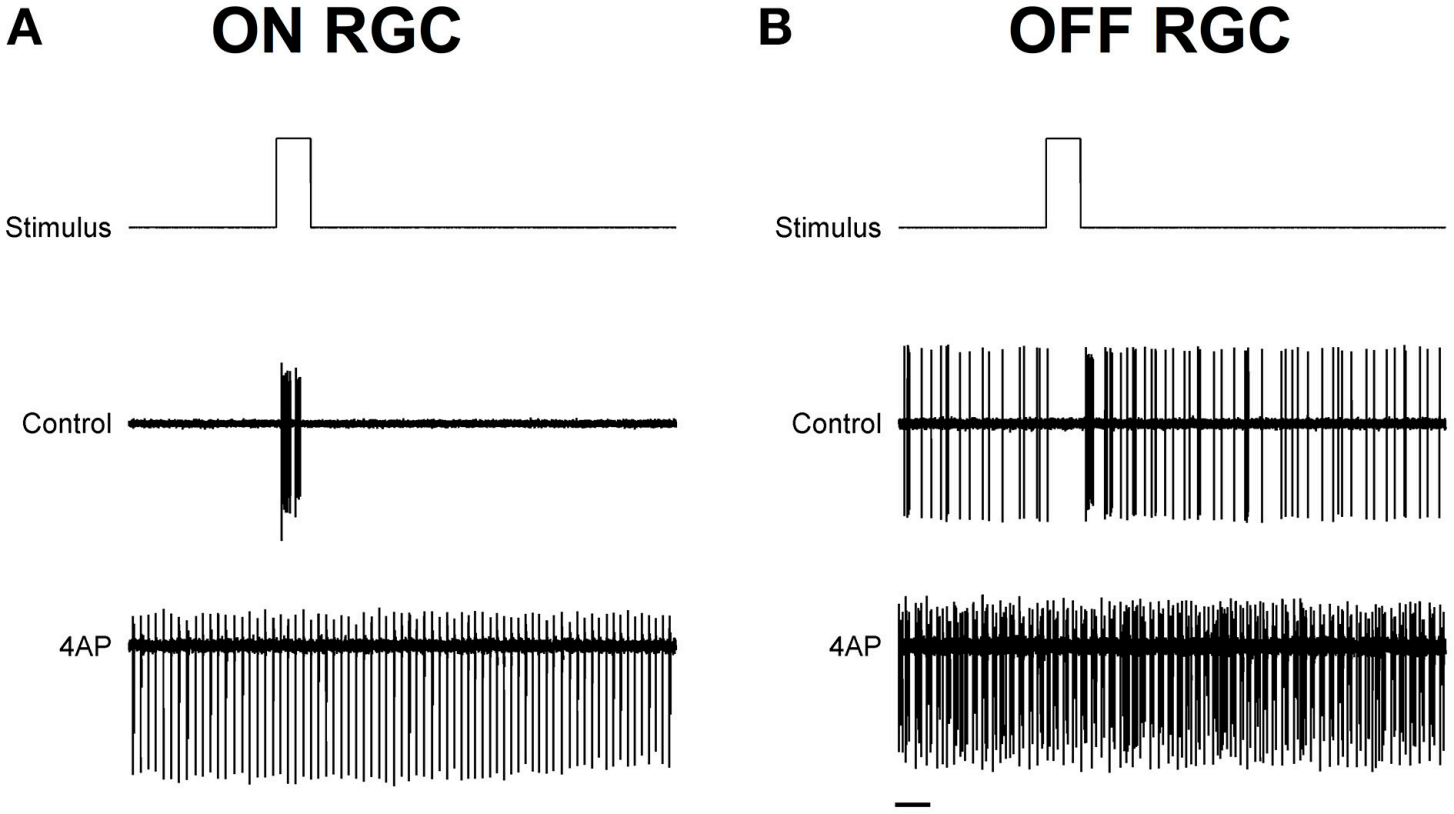
**Exemplar light induced ON and OFF responses in rat RGCs**. **(A,B)** Spike recordings and the effect of light pulses (1 s) in two exemplar ON **(A)** and OFF **(B)** RGCs. Top lines are the schematic representation of the light stimulus. The middle and bottom traces are representative spike recording traces before (Middle) and during (bottom) the application of 4-AP (0.02 mM). Notice how the application of 4-AP increases spontaneous activity and reduces the stimulus dependent variation in the cell activity. Black scale bar is equal to 1 s.

**Figure 3:**
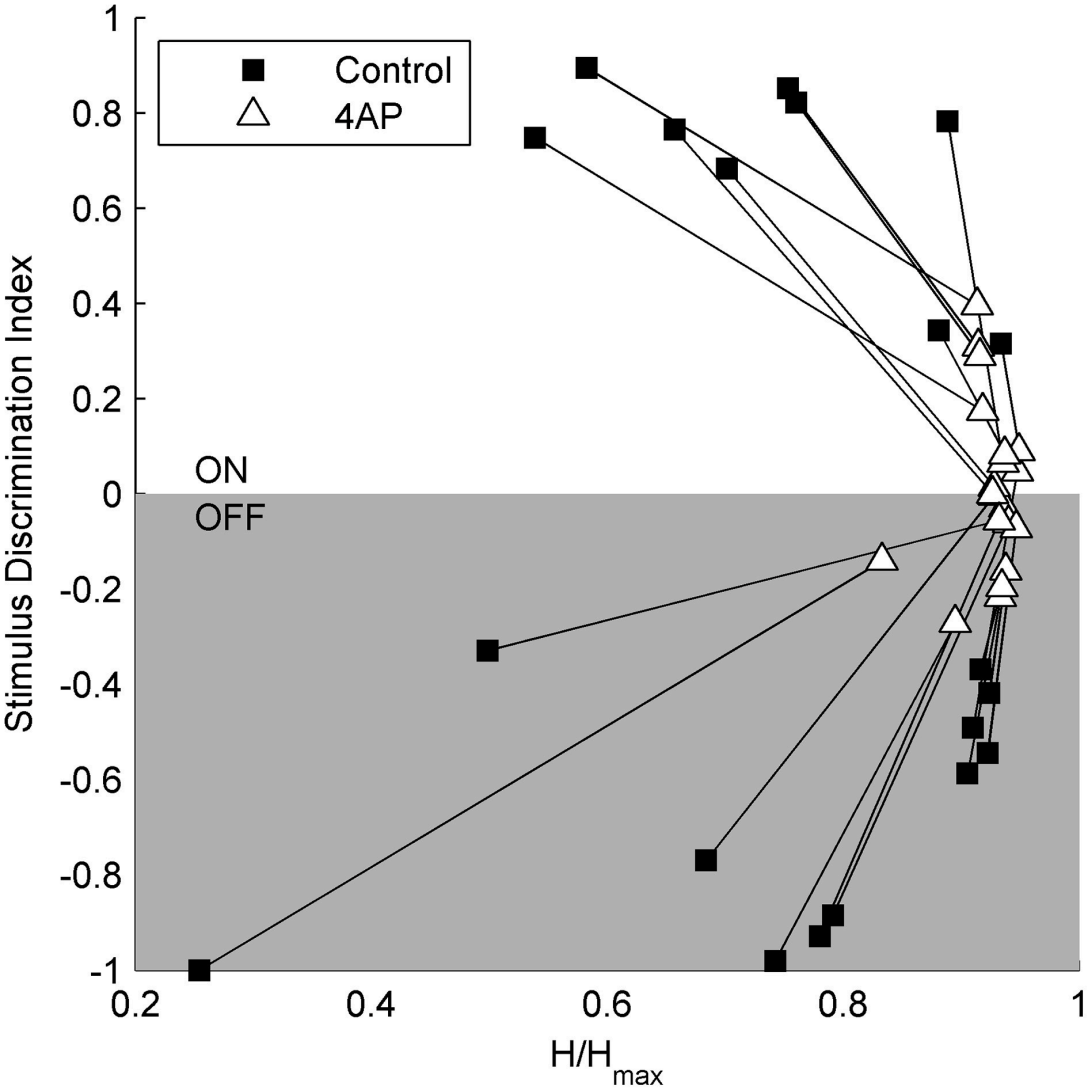
**Relation between stimulus selectivity and spike rate variability suggests a reduction of the visual transfer of information in the presence of 4-AP**. Stimulus selectivity (y-axis) is expressed as a *Discrimination Index = (A1 – A2)/(A1 + A2)*, where A1 is the number of spikes during the light pulse and A2 is the number of spikes right after the stimulus calculated on a time window of the same duration as the light pulse. It is positive for ON cells and negative for OFF cells. In both ON and OFF the Discrimination Index converges to 0 following the application of 4-AP suggesting a firing activity which becomes less dependent on light stimuli. The entropy of ISIs (x-axis) was calculated as *H* = $\underset{\text{i}}{\sum }\left({\text{ISI}}_{\text{i}}∕\underset{\text{j}}{\sum }{\text{ISI}}_{\text{j}}\right)*\text{log}\left({\text{ISI}}_{\text{i}}∕\underset{\text{j}}{\sum }{\text{ISI}}_{\text{j}}\right)$ on a perisimuls time window of 4 s. The Standardized Entropy (H_s_) was then obtained dividing H by the maximum entropy value of each ISI series [calculated as *log(n)* where *n* is the number of ISIs] to account for differences in the number of spikes among the series. In accordance to the previous index, the entropy is much closer to its maximum value after the application of 4-AP. All calculations where performed on single traces obtained for each cell by the superimposition of 30 sweeps, in both control and 4-AP conditions. Pairs of black squares and white triangles connected by lines indicate single RGCs before (black squares) and in the presence of 4-AP (white triangles).

To test the light evoked response, the isolated retinas were stimulated with full field light pulses (1 s pulses) repeated every 20 s (30 pulses for each experimental condition). Based on these data, we could classify cells based on a Discrimination Index (see Materials and Methods). Cells with a positive discrimination index (meaning they were more likely to respond to sudden increase in luminance) were classified as ON ganglion cells (*n* = 9), while cells with a negative discrimination index (meaning they were more likely to respond to sudden decrease in luminance) were classified as OFF ganglion cells (*n* = 11). No biphasic ON-OFF cells were encountered during this study. Although the application of 4-AP increased spontaneous activity, it reduced the stimulus dependent variation in cell activity, i.e., cells were less able to distinguish between dark and light (Figure 2). Indeed the discrimination index was generally closer to 0 both in ON and OFF cells after the application of 4-AP, meaning that cells were equally likely to fire during their appropriate stimulus and without any stimulation (Discrimination Index Mean Absolute Deviation from 0: 0.68 in Controls, 0.13 with 4-AP, *p* < 0.01 calculated using a logistic model as described in the Materials and Methods Section, Figure 3). As the discrimination was reduced, the standardized entropy Hs (see Materials and Methods) of the Interspike Intervals (ISIs) was closer to its maximum value, suggesting a reduction in the information transfer (mean Hs before 4-AP = 0.75, STD = 0.18; mean Hs after 4-AP = 0.93, STD = 0.03; Figure 3).

### Selective suppression of ON-OFF thalamo-cortical responses by 4-AP and L-AP4

The second step was to assess the efficacy of the two drugs used throughout the study when administered to live animals with intravitreal injections.

**Figure 4:**
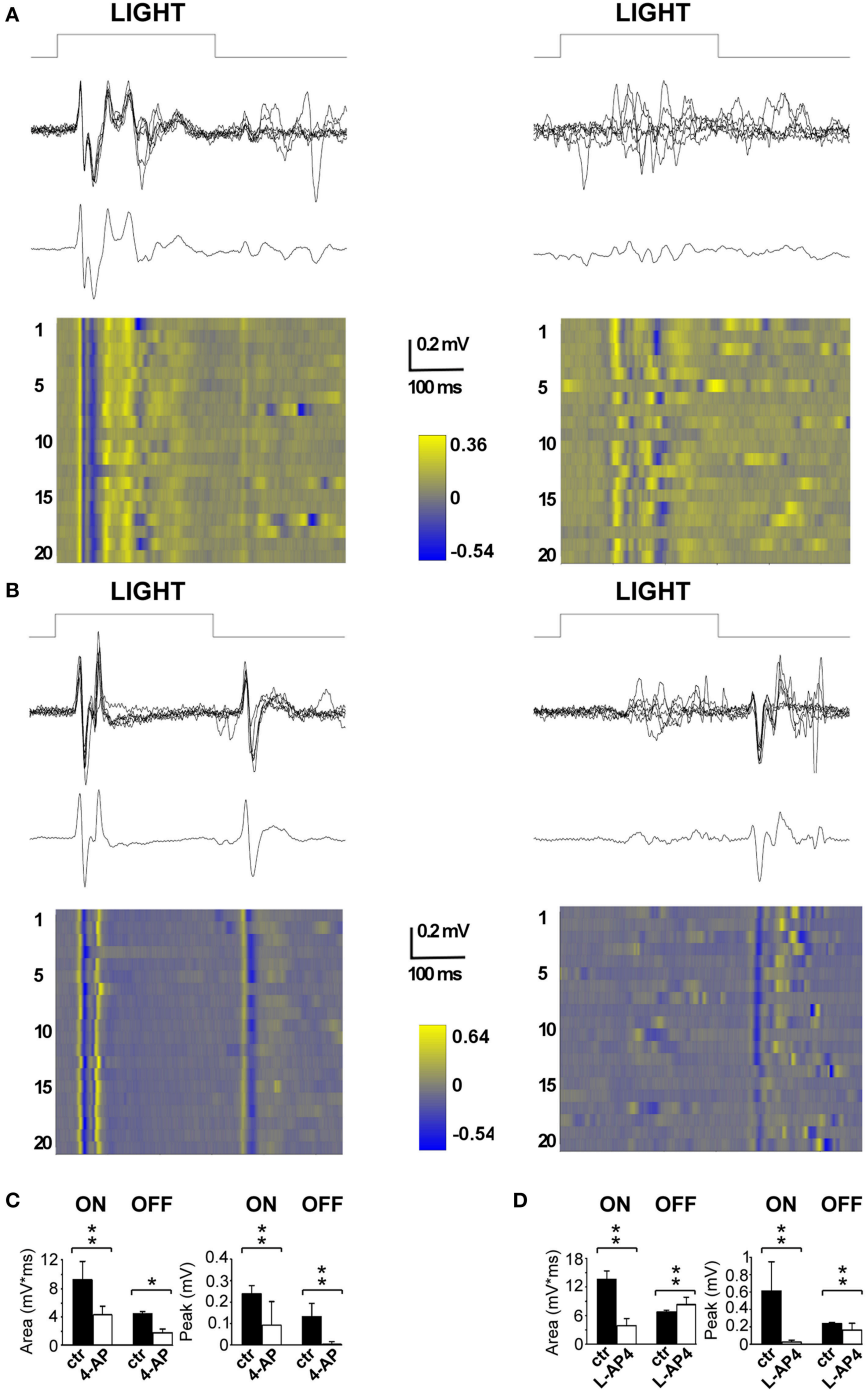
**Effects of 4-AP and L-AP4 on visual evoked potentials (VEPs) recorded from the V1 area**. **(A,B)** Representative VEPs recorded before (left) and after (right) the application of 4-AP (2 mM) **(A)** and L-AP4 (2 mM) **(B)**. Rats were stimulated with light pulses lasting 300 ms (111 μW/cm^2^) before (traces on the left) and after (traces on the right) the intravitreal injection of either 4-AP **(A)** or L-AP4 **(B)**. In both panels, above it is plotted the superimposition of *n* = 6 individuals sweeps, below the corresponding ensemble average. Notice how in control conditions (traces on the left) after the change in luminance there is a clear change in voltage (ON cortical response) and that the interruption of the stimulation also produces an effect which is smaller and less reproducible in shape across different experiments (OFF cortical response). **(C,D)** Quantitative analysis for the ON and OFF responses before (black bars) and after (white bars) intravitreal injections of 4-AP **(C)** and L-AP4 **(D)**. Bars plot the integral of root mean square or quadratic mean (RMS) (right bars in **C,D**) or the peak amplitude of the first positive response (left bars in **C,D**). The RMS of the ON and OFF responses were integrated for 150 ms from the start (ON) or from the end (OFF) of light pulses. All pair comparisons yielded significant differences (*n* = 3 rats for each condition; 60 sweeps before and after drug treatment; stars indicate significance: ^*^indicates *p*-values < 0.05, ^**^indicates *p*-values < 0.01; permutation tests based on *t*-type statistics).

The first drug we tested was 4-AP; specifically we wanted to examine the *in vivo* effects of the circuit alterations measured on the isolated retina. The second drug tested was L-AP4, a mGlurR-6 agonist known to block the activity of ON bipolar cells (Slaughter and Miller, 1981). In the latter case, the rationale was to obtain a single pathway output from the retina, in contrast with the uncoordinated ON-OFF RGC activity produced by 4-AP. To assess the efficacy of intravitreal injection of the selected drugs, we recorded visual evoked potentials (VEPs) induced by flash stimulation before and after the intravitreal injection to obtain internal comparisons and to minimize the effects of inter animal variability among recordings. In these experiments, we sampled V1 activity on anesthetized and ventilated animals (see Materials and Methods for details). As depicted in Figure 4, a repeated stimulation with 300 ms light flashes in control conditions produced a very clear and reliable response across successive trials, whose amplitude and waveform was preserved over time (stimulation rate 0.1 Hz; flash intensity 111 μW/cm^2^) (left traces in Figures 4A,B). The early biphasic ON-response peaked on average at 40 ms (P wave) and 50 ms (N wave) from the beginning of the light stimulus. Overall the ON response lasted from 30 to 200 ms. In most cases a clear OFF-response could also be detected. The initial positive P wave of the OFF response peaked at 70 ms from the end of the light stimulus. When 4-AP was administered via intravitreal injection (2 mM; eye contralateral to the V1 recording site) it clearly reduced the detectable visual cortex responses seen in control conditions. The early phase of the ON and OFF responses almost completely disappeared leaving just some highly variable asynchronous activity (*p* < 0.05; Figure 4A, right). On the contrary, when the selective group III metabotropic glutamate receptor agonist L-AP4 was tested, we observed, as expected, a selective inhibition of the ON response, contralateral to the injected eye, (*p* < 0.05; Figure 4B) while the OFF response was still clearly present. Figures 4C,D shows the quantitative results across all experiments to illustrate the important reduction of the entire ON and OFF waveform by 4-AP, while L-AP4 essentially spared the OFF response, with a small, although statistically significant, change in the shape and duration of its waveform.

### Effects of ON-OFF visual patterns on dLGN activity

For this experiment, rats were subdivided in two experimental groups, the no-light and the light-stimulated groups. Light stimulated animals received monocular light stimulation with 2 Hz reversal, at constant overall luminance, of black and white reversing vertical stripes (0.5 cycle/degree; left eye stimulation) (see Materials and Methods). To study the distribution of active neurons in the visual thalamus, we used the activity-dependent labeling method based on the expression of the c-Fos gene protein product (Sheng et al., 1990; Flavell and Greenberg, 2008) on 10× magnification images of the dLGN. Active neurons, independently of their level of c-Fos activation, were grouped together in a single class (c-Fos positive), which was distinct from the inactive cell group (c-Fos negative) (see Materials and Methods for distinction). Animals were dark reared for 48 h before the experiment to reduce the basal level of c-Fos expression in thalamic neurons. In the no-light condition, very spare active cells could be identified inside the dLGN, the image-forming subregion of the LGN structure (Table 1). Following monocular ON-OFF light stimulation, the number of active cells in the dLGN did not change significantly with respect to the no-light condition (Table 1, *p* > 0.05; see Supplementary Figure 3). No significant differences were also found between contralateral and ipsilateral LGNs (*p* > 0.05). Since ON-OFF light stimulations induced a very clear activation in the matching retinas (Table 2), these results might suggest the presence of some kind of inhibitory mechanisms counteracting a strong activation of postsynaptic dLGN cells, preventing a detectable expression of c-Fos.

**Table 1:** **NeunN+/c-Fos+ cell counts (cells/mm^2^) in the dLGN for the conditions tested**.

	**Darkness**	**Grid**	**Darkness**	**Grid**	**Rats**	**Slice/Rat**
	***Contralateral***		***Ipsilateral***			
Control	45 (±34)	47 (±17)	45 (±34)	38 (±20)	8	9
4-AP	151 (±28)	228 (±45)	35 (±30)	40 (±20)	8	9
L-AP4	63 (±44)	442 (±60)	41 (±25)	35 (±21)	6	9

*Table reports cells counts (cells/mm^2^) of c-Fos positive cells among NeuN positive cells in the dLGN (contralateral and ipsilateral to the tested eye) for various conditions. Notice the different behavior of the 4-AP group compared to the L-AP4 group: in the first no differential activation could be detected between visual stimulation and darkness condition, while in the latter a clear increase in c-Fos expression could be detected after visual stimulation. The fifth column reports the number of animals tested for each pharmacological manipulation, equally subdivided between darkness and visual stimulation. The last column reports the number of slices analyzed per each animal*.

**Table 2:** **NeunN+/c-Fos+ cell counts (cells/mm^2^) in the retina for the conditions tested**.

	**Darkness**	**Grid**	**Rats**
Control	31 (±6)	138 (±41)	8
4-AP	120 (±69)	160 (±56)	8
L-AP4	161 (±107)	96 (±58)	6

*Table reports cells counts (cells/mm^2^) of c-Fos positive cells among NeuN positive cells in the retina for various conditions. Notice how, even in the control group, a clear increase in c-Fos positive cell density could be found after visual stimulation, as opposed to the dLGN behavior in the same conditions. The last column reports the number of animals tested for each pharmacological manipulation, equally subdivided between darkness and visual stimulation*.

### Effects of concomitant ON and OFF RGCs activation by 4-AP on dLGN activity

**Figure 5:**
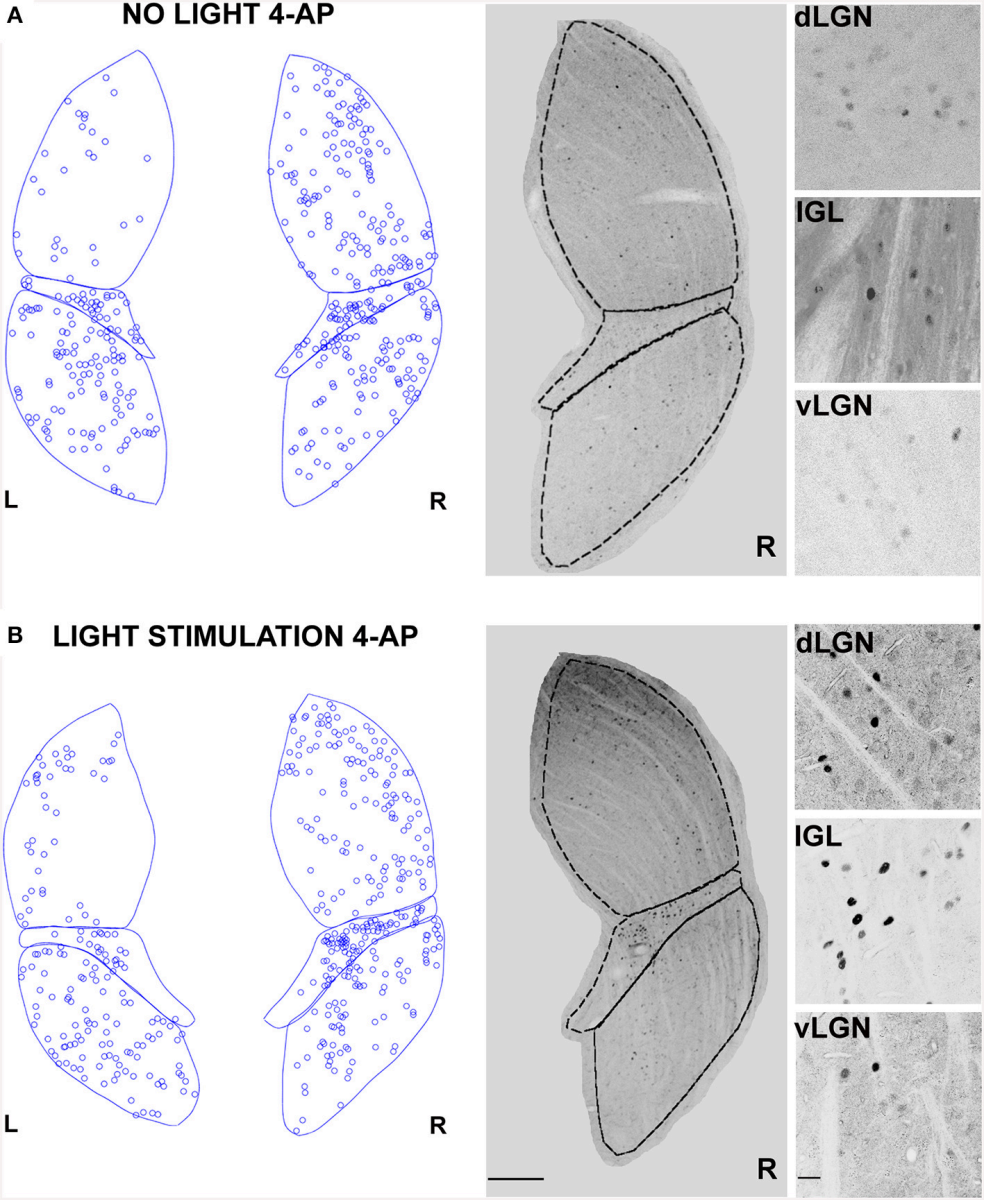
**Changes in LGN activity by intravitreal injection of 4-aminopyridine (4-AP)**. **(A,B)** Representative immunostainings (right) and digital reconstructions (left) of the right (R) and left (L) LGNs (same coronal sections). Circles report the location of identified c-Fos positive cells (for segmentation algorithm, see Materials and Methods). In these experiments the A-type potassium channel blocker 4-aminopyridine was injected in the left eye vitreal chamber (2 mM). **(A)** An exemplar result to illustrate the effect of 4-AP on c-Fos activation pattern in the no-light condition (animal kept in darkness). **(B)** The effect of ON-OFF light activation in the presence of 4-AP. Light-stimulation was obtained with alternating black and white vertical bars at constant overall luminance (white bars 37 mW/m2; black bars 0.11 mW/m2; 2 h; 2 Hz refresh rate; 0.5 cycle/degree; left eye stimulation). The three small panels on the right are magnification of the dLGN, IGL, and vLGN from the corresponding sections. Continuous and dashed lines indicate edges of dLGN, IGL, and vLGN. Notice how while 4-AP increases the basal activation of the dLGN (see data in the text, also compare L and R), the effect of light stimulation on this structure is not noticeable (Compare R in **A** with **B**). Calibration bar is 200 μm for the large immunostaining panels and 50 μm for the small insets.

We showed that 4-AP can produce a random chaotic activation of RGCs shunting the upstream retinal circuit regulation. To test the effect of concomitant random activation of the ON and OFF pathways on thalamic c-Fos expression, we injected 4-AP in one eye (2 mM). Following the application of 4-AP, in darkness conditions, we observed a ~4 fold increase in the number of active RGCs in the retina (Table 2). As presented in Figure 5A, the injection 4-AP increased the number of active neurons in the contralateral dLGN with respect to animals kept in darkness in control conditions (Table 1, *p* < 0.05). The ON-OFF light-stimulation after the injection of 4-AP produced a clear activation of the contralateral dLGN as compared to the visual stimulation alone (*p* < 0.05) but most of this effect was not visually driven. In fact, in the 4-AP experimental group, the ON-OFF light stimulation produced a small and not statistically significant increment in the number of activated cells in the retina (33%) and dLGN (51%) with respect to the no-light condition (Figures 5A,B) (*p* = 0.32). No significant activation of the ipsilateral dLGN could be detected. In these 4-AP experiments, the distribution of active neurons in the contralateral dLGN was not homogeneous, with an increased density of c-Fos positive cells near the lateral edge (see below). This contrasted with a homogeneous distribution of neurons across the dLGN structure (staining for the neuronal marker NeuN Mullen et al., 1992; Kim et al., 2009; see below and **Figure 9**).

### Effects of ON retinal pathway blockade by L-AP4 on dLGN activity

**Figure 6:**
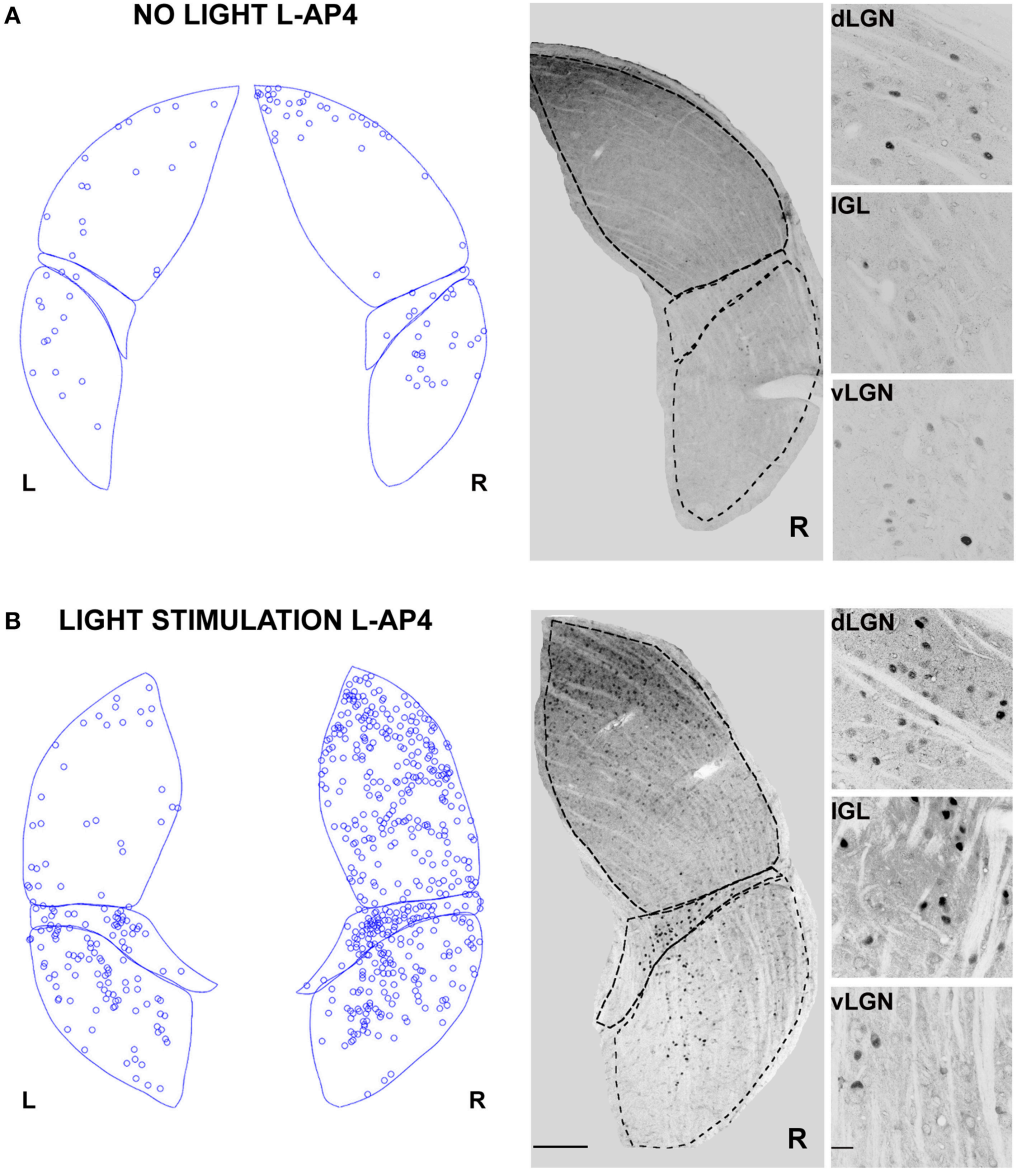
**Changes in LGN activity by intravitreal injection of the ON- pathway inhibitor L-AP4**. **(A,B)** Representative immunostainings to illustrate the location of c-Fos positive nuclei when L-AP4 is injected in the left eye vitreal chamber (2 mM). **(A)** The effect of L-AP4 on the pattern of c-Fos activation in the ipsilateral (L) or contralateral (R) LGN for an animal kept in darkness. **(B)** An exemplar LGN section to illustrate the clear enhancement by the mGluR6 agonist L-AP4 of the light induced response by ON-OFF light activation. Notice how the c-Fos activation of the IGL remains unaffected by the drug. This presumably reflects the predominant input from melanopsin-containing intrinsically photosensitive retinal ganglion cells in the non-image forming pathway (Güler et al., 2007). Small panels on the right are magnification of the dLGN, IGL, and vLGN from the corresponding sections. Calibration bar is 200 μm for the large immunostaining panels and 50 μm for the small insets.

In contrast with the previous experiment, we wanted to test the effect of a pure single channel (OFF) activation on the c-Fos expression in dLGN. To obtain such selective activation, we intravitreally injected L-AP4, known to inhibit the activation of ON-bipolar cells (Slaughter and Miller, 1981), sparing the OFF retinal pathway. This treatment produced little neuronal activation in both the retina and the LGN in the absence of a visual stimulus if compared to the darkness in control conditions (Table 2; *p* > 0.05) (Figure 6A) but a clear recruitment could be driven by visual stimulation both in the retina (~600%, Table 2) and in the dLGN (Figure 6B and Table 1, *p* < 0.01). The presence of a visually driven response and the degree of dLGN recruitment seen with L-AP4 injection followed by visual stimulation clearly differed from the results obtained with 4-AP injection. The strong light induced response in a condition where the activity of the ON channel is suppressed confirms previous results on tonic inhibition of the OFF bipolar cells by the ON pathway (Zaghloul et al., 2003; Margolis and Detwiler, 2007). No significant activation of the ipsilateral dLGN could be detected. As after the injection of 4-AP, active thalamic neurons appeared to cluster near the lateral edge of the dLGN (see later). This result suggests that the lateral border of the dLGN, irrespectively of the ON and OFF modality, concentrates neurons with either the highest firing activity or with the lowest inhibitory input.

### Selective recruitment of dLGN GABAergic interneurons

**Figure 7:**
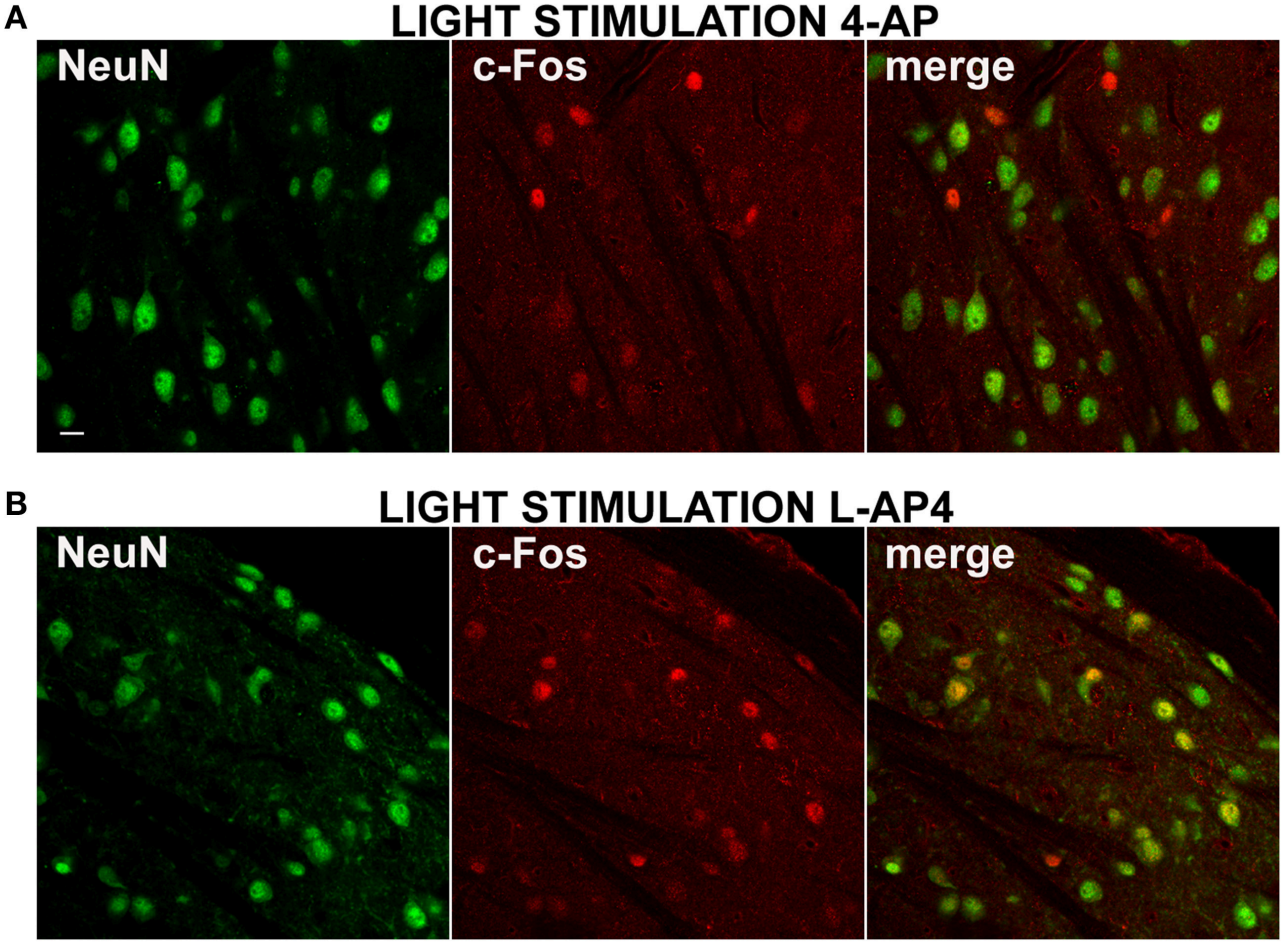
**Differential activation of NeuN positive neurons by 4-AP and L-AP4**. **(A,B)** Double immmunostaining for the neuronal marker NeuN (left) and the activity marker c-Fos (middle) and the merge image (right) in the dorsal portion of the lateral geniculate nucleus. Rats were injected into the eye with 4-AP (2 mM; **A**) and L-AP4 (2 mM; **B**). In **(A,B)** dLGN NeuN and c-Fos images were obtained from the same optical section of contralateral hemisphere. Notice how with 4-AP, almost all c-Fos positive cells are NeuN negative or weakly positive. The opposite is seen with L-AP4, where most active cells are NeuN positive. Magnification, 63×. White calibration bar, 10 μm.

In all experiments presented up to now, dLGN cells were identified by counterstaining for the neuronal marker NeuN (1704 ± 505 NeuN positive cells/mm^2^, mean ± SD; *n* = 38 dLGN sections) (Mullen et al., 1992). From preliminary inspection, we realized that many active cells in the 4-AP experimental group, most likely neurons because of their morphological shape (based on interference contrast imaging), were either unstained or very faintly stained with antibodies against the NeuN antigen (Figure 7A). When datasets of c-Fos positive cells were subdivided into NeuN positive and negative cells (threshold for inclusion = background fluorescence noise + 3 times its SD), 4-aminopiridine with or without monocular light stimulation was found to activate mainly NeuN negative cells (73 ± 25% of active cells were NeuN negative; mean ± SD; *n* = 30 dLGN sections). These active cells were usually surrounded by a large number of inactive NeuN positive cells (Figure 7A). On the contrary, in the L-AP4 treated group, light recruited mainly neurons expressing high levels of NeuN protein (65 ± 11% of active cells were NeuN positive; mean ± SD; *n* = 32 dLGN sections) (Figure 7B). Although these results are in contrast with the general idea that the nuclear antigen NeuN, corresponding to the protein product of the Fox-3 gene (Kim et al., 2009), is a reliable marker for neurons, it has been reported that, in some brain areas, *bona fide* neurons do not stain with antibodies against NeuN (Mullen et al., 1992; Weyer and Schilling, 2003): in the cerebellum these cells coincide with well-known GABAergic neurons (Weyer and Schilling, 2003).

**Figure 8:**
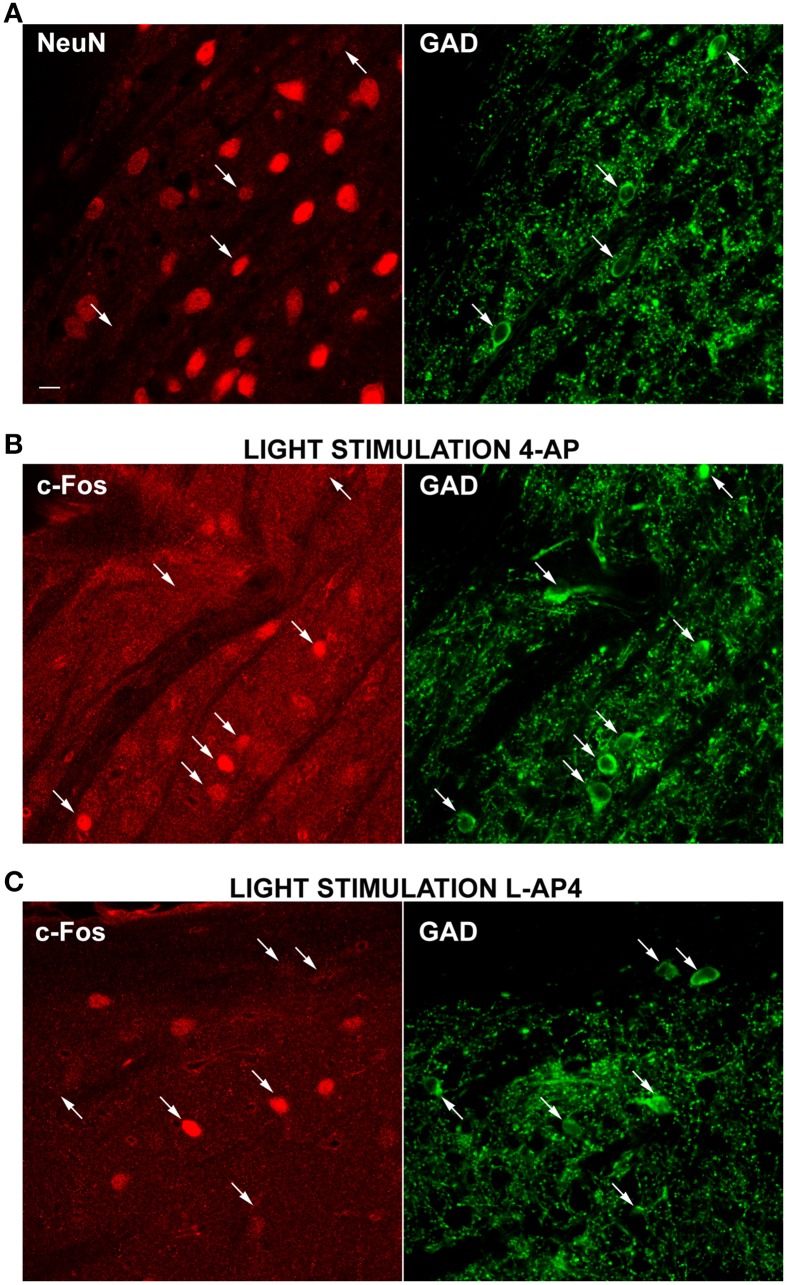
**Differential activation of GAD positive neurons by 4-AP and L-AP4**. **(A–C)** Double-immunolabelings of the dLGN for the neuronal cell marker NeuN (left) and GAD (right), the synthesizing enzyme of GABA **(A)** and for the activity marker c-Fos (left) together with GAD (right) **(B,C)**. A mix of GAD65 and 67 antibodies was used to better visualize GABAergic neurons. Notice how a strong GAD fluorescence is present in the somatic cytosol, whereas nuclei are unstained. Puncta are the dendritic and axonal arborisations of these interneuronal cells. In Panel **A**, arrows point to some exemplars GABAergic neurons which are in most cases either NeuN negative or weakly positive (3 out of 4 cells). Images presented **(B,C)** were obtained from the dLGN of animals injected into the eye with 4-AP (2 mM; **B**) and L-AP4 (2 mM; **C**) and stimulated contralaterally with ON-OFF light patterns. Notice the differential activation of GAD positive cells in the two experimental conditions. With 4-AP **(B)**, of the seven GAD positive cells indicated by arrows, five are strongly c-Fos positive **(C)**, of the six GAD cells indicated by arrows, only two are active. Magnification, 63×. White calibration bar, 10 μm.

Therefore, to clarify this issue and determine if NeuN negative cells could belong to a population of GABAergic interneurons, we double-immunolabeled dLGN cells with a mixture of antibodies against the GABA synthesizing enzymes (GAD65 and GAD67) together with antibodies against the NeuN antigen. As depicted in Figure 8A, the GAD positive cells were fewer than the NeuN positive cells (278 ± 140 GAD positive cells/mm^2^, mean ± SD; *n* = 49 fields) and most of these cells were either devoid or very weakly counterstained for the neuronal antigen NeuN (this observation was not a general finding in all brain areas, see reticular nucleus staining in Supplementary Figure 4). GAD staining does not allow single cell identification at 10× magnification. Therefore, we used 63× magnification to locate GAD positive cells and determine the percentage of inhibitory neurons stained with NeuN antibody. We found that 53.3% of the GAD positive cells showed very faint or no staining for NeuN (*n* = 272 cells, 107 fields, 15 slices from 5 animals).

Then, we run a second set of experiments to test the main hypothesis that 4-AP injection was more effective in recruiting inhibitory interneurons than L-AP4 injection. A total of six animals were injected (three with with 4-AP and three with L-AP4) and then exposed visual stimulation. dLGN sections were then double stained for c-Fos and GAD and the activation of inhibitory neurons analyzed again at 63× magnification (see Table 3). We found that 64 and 33% of GAD positive cells expressed high c-Fos levels, in the 4-AP and L-AP4 experimental groups respectively (see Table 3, *p* < 0.001; see Supplementary Figure 2 and Materials and Methods for threshold selection and statistics) (Figures 8B,C). These results suggest that the preferred phenotype of activated neurons differed significantly between these two activation modalities. L-AP4 elicits a weaker response of inhibitory interneurons, thus yielding a larger c-Fos expression in the dLGN, mostly by NeuN positive neurons. On the other side, 4-AP is able to achieve a stronger recruitment of inhibitory interneurons, and this reflects in the smaller number of overall active neurons between 4-AP and L-AP4 treatments with light stimulation (although presumably because of the high variability in cell counts and sample size this reduction did not reach the significance level, *p* = 0.09).

**Table 3:** **GAD+, c-Fos+, and GAD+/c-Fos+ cell counts (cells/field) at 63× magnification in the dLGN after visual stimulation and intravitral drug injection**.

**Treatment**	**Rat**	**Slices**	**cFos+ Cells**	**GAD+ Cells**	**GAD−/cFos+ Cells**	**cFos+ per Field**	**GAD+ per Field**	**GAD+/cFos+ per Field**
4-AP	3	8	351	123	79	7.313	2.563	1.646
L-AP4	3	10	642	162	54	8.447	2.132	0.711

*Table reports cells counts (cells/field) of GAD positive (column 3), c-Fos positive (column 4), and double GAD/c-Fos positive cells (column 5) in the dLGN following visual stimulation and intravitreal drug injection. All cells were identified at 63× magnification to allow precise detection of GAD positive cells. Highly c-Fos expressing cells (refer to text for selection threshold) were identified as c-Fos positive cells. The last three columns represent the same counts normalized by the number of fields analyzed in each condition. The first column report the number of animal tested in each condition, the second column reports the total number of slices analyzed per condition (multiple fields were analyzed for each slice)*.

We also analyzed the spatial distribution of GAD positive and GAD/c-Fos positive cells. To this aim we acquired high magnification (63×) images in nine separate fields per each slice. These fields were distributed along both the lateral-medial axis and the dorso-ventral axis (lateral, central, medial positions combined with superior, central and inferior positions). Each field position was then recorded and GAD positive cells, c-Fos positive cells and double positive cells counted. Regarding the distribution of GAD cells, when number and density of these cells were counted in each one of these locations, repeating this for 83 different areas, by using a generalized linear model with a Poisson error distribution, we could test the null hypothesis of homogeneous cell counts across all different locations. With this analysis no evidence for divergence from the null hypothesis could be found (*p* always above 0.48) confirming both our visual impression and previous findings (Gabbott and Bacon, 1994) that the density of GABAergic cells does not change along the superior-inferior and lateral-medial axes of the dLGN. Then we estimated the probability that a GAD positive cell at a given lateral-medial location was also expressing c-Fos at high level (same threshold used above). We found, by using a logistic regression, that highly active GABAergic interneurons were localized closer to the lateral edge (*p* = 0.033).

### Statistical analysis of the spatial distribution of active neurons

**Figure 9:**
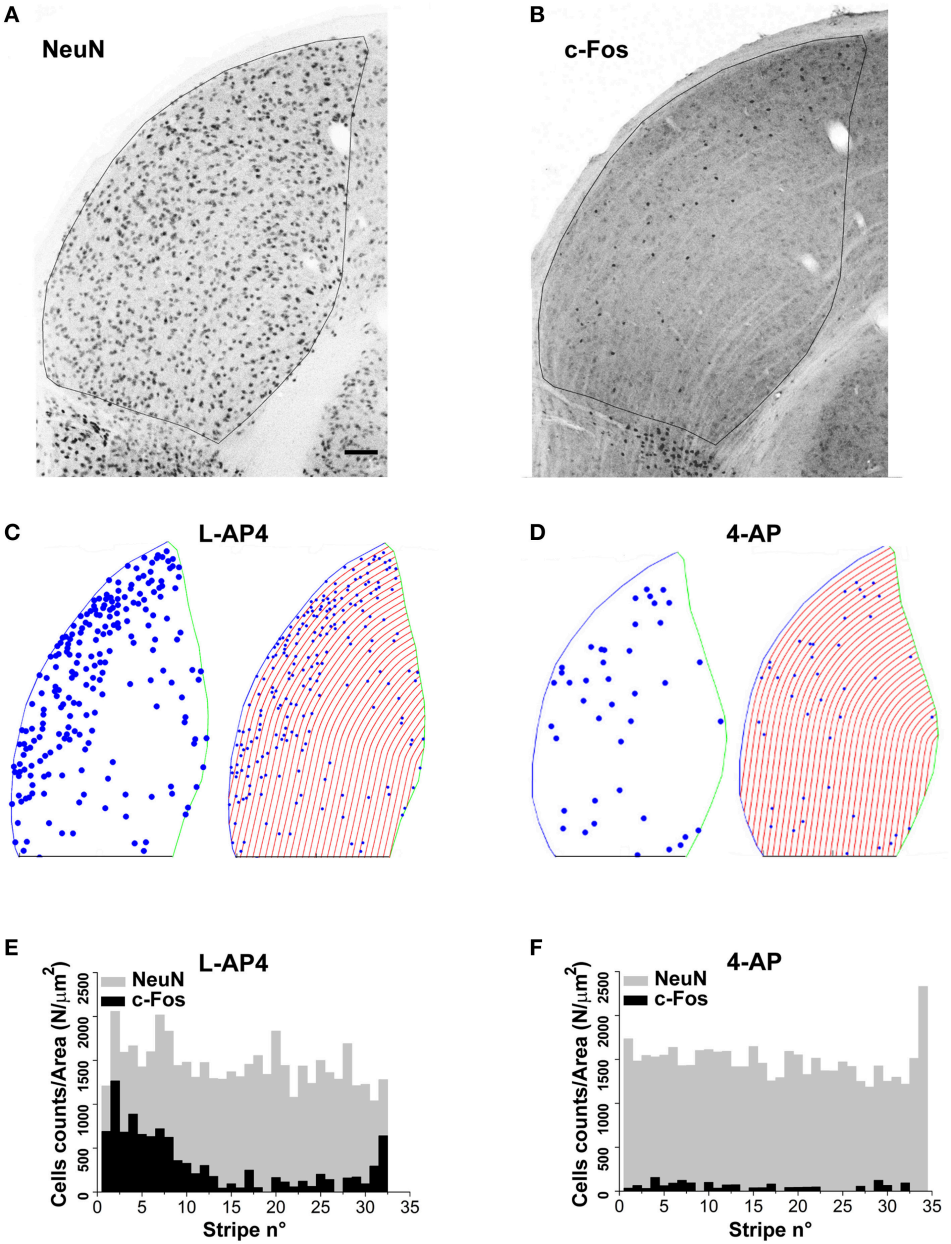
**Lateralization of active NeuN positive neurons in the dLGN structure**. **(A,B)** Double immunostaining for the neuronal marker NeuN **(A)** and the c-Fos protein **(B)** in the dorsal portion of the right lateral geniculate nucleus. These two panels refer to an animal whose left eye was injected with 4-AP (2 mM) and then light stimulated. **(C,D)** Digital reconstructions to illustrate the location of double positive NeuN-c-Fos neurons and the procedure used to subdivide the dLGN structure in parallel stripes of constant thickness (see Materials and Methods Section for the procedure used to subdivide the LGN in stripes). The panels illustrate two exemplar experiments where the left eye was injected with either L-AP4 (2 mM; **C**) or 4-AP (2 mM; **D**) and then light stimulated. **(E,F)** Histograms plot the number of NeuN positive cells (gray bars) and double positive NeuN-c-Fos neurons (black bars) in each stripe. The histograms plot raw cell counts for sequential stripes from a single section normalized by the corresponding stripe area to compensate for the reduction of stripe area due to the LGN curvature. Histograms of normalized counts always displayed a fairly uniform distribution of NeuN cells along the latero-medial axis (*p* > 0.4) while double positive NeuN-c-Fos neurons always displayed a skewed distribution with higher density for stripes near the lateral edge of the dLGN. To test if the median value of double positive NeuN-c-Fos neurons was significantly smaller (closer to the lateral edge) than the median value of the NeuN cell population, for each dLGN section 10.000 random samples were extracted from the NeuN set (sample size equal to the observed NeuN-c-Fos sample size). Then, for each sample (*n* = 2 randomly selected sections from each animal; *n* = 3 animals for each experimental condition) the position of each cell along the lateral-medial axis was calculated as before and the median value for each sample calculated. The *p*-value was calculated as described in theMaterials and Methods Section with Monte Carlo methods. *P*-values where then adjusted for multiplicity by means of the Bonferroni-Holm procedure. Results indicate that in every case the null hypothesis (double positive neurons median value not smaller than NeuN set median value) could be rejected (*p* always < 0.05 using Bonferroni-Holm correction).

The results presented up to now suggest two different modalities of dLGN activation in the presence of either 4-AP or L-AP4. We therefore asked the question if these differences correlated with a different distribution of active neurons over this thalamic sub-region. Upon visual inspection, the c-Fos activation pattern showed a non-homogenous spatial distribution of activated neurons with more active cells located on the lateral edge of this structure. To study this feature in deeper detail, we analyzed the spatial distribution of activated (c-Fos positive) cells identified as neurons based on their staining for the neuronal marker NeuN (10× magnification images). Based on the comparative observation of LGN sections by fluorescence and interference contrast imaging, NeuN positive cells represent the large majority of neuronal cells that could be bona fide identified in the dLGN structure. To estimate the spatial randomness of activated neurons (NeuN positive), we analyzed the distribution of c-Fos positive cells in those experiments where 4-AP or L-AP4 treatments were associated with ON-OFF light stimulation. dLGN sections were reconstructed and subdivided using the Voronoi tessellation method based on the point pattern of double NeuN/c-Fos positive cells (data not shown). Individual areas of the Voronoi polygons, each one associated to an identified neuron belonging to a single dLGN slice, were used to calculate the entropy value of the experimental activation pattern. In this analysis the null hypothesis was that the observed pattern of activated cells was not different from a random activation of the NeuN data set (the *p*-value was estimated by Monte Carlo methods, see Materials and Methods Section). In L-AP4 injected animals, the spatial pattern of activated cells was found significantly different from a random extraction from the corresponding NeuN data set in every dLGN section analyzed (*n* = 6; *p* always < 0.001). For 4-AP experiments the same null hypothesis could not be rejected in two out of six cases (significance level at 95%). The latter result could be explained either by a more homogeneous activation of the NeuN set when the A-type potassium channels blocker 4-AP is used or by some intrinsic limitation of the test procedure due to a reduced sample size of activated neurons under this experimental condition. To further evaluate this issue, and clarify if the observed inhomogeneity could be partially attributed to a subtle increase in cell density in the lateral portion of the dLGN, we subdivided this structure into lateral-medial stripes with a constant thickness, drawn parallel to the dLGN lateral edge (See Materials and Methods). For every stripe, we computed the frequency of NeuN positive neurons and activated cells. The pictures and histograms presented in Figure 9 depict the frequency of NeuN positive and NeuN/c-Fos double positive neurons for individual stripes. In agreement with the results presented above, NeuN positive neurons showed a uniform spatial distribution when their frequency was corrected by the individual stripe area (the null hypothesis of uniformity could not be rejected with *p*-values always > 0.4). In order to test whether c-Fos positive neurons could simply be a random activation of the uniform NeuN neuronal population, from each available slice, by Monte Carlo Sampling we extracted *N* = 10.000 random samples from the NeuN data set, with a sample size corresponding to the experimental number of activated cells in each slice. The distribution of the median values of these random samples was used as an estimate of the test distribution under the null hypothesis. The results lead to a clear evidence of lateralization of activated neurons in the dLGN structure for both L-AP4 and 4-AP groups (*p*-values always < 0.05, Bonferroni-Holm correction). This suggest that despite the crossed retino-thalamic input reaching both medial and lateral LGN districts, the lateral border concentrate most of the activity behaving as a sort of functional stripe.

## Discussion

In this study we characterized the effects of two different (chaotic or highly coherent) retinal inputs to the dLGN in terms of different patterns of c-Fos expression in this nucleus. As a reporter of activity, we used the expression of the early gene c-Fos paired with specific labeling of neuronal cells (Sheng et al., 1990; Murphy et al., 1991, 2004; Montero and Jian, 1995; Correa-Lacarcel et al., 2000; Greferath et al., 2004; Lu et al., 2004; Flavell and Greenberg, 2008; Dai et al., 2009). Despite the fact that some anatomical features of lamination have been previously described in the rat visual thalamus, with segregation of ipsilateral and contralateral RGC axon terminals (Godement et al., 1980, 1984; Reese, 1988; Huberman et al., 2008, 2009; Kim et al., 2010; Rivlin-Etzion et al., 2011), little information is available on the spatial and functional organization of cells and synapses across the dLGN structure. In the past, the analysis of visually driven c-Fos activation in the rat thalamus has provided evidence for some recruitment of neuronal cells in the dLGN (Montero and Jian, 1995; Correa-Lacarcel et al., 2000; Lu et al., 2004). In none of these studies a functional lamination of the dLGN response was reported, presumably because the integrated firing activity in the thalamus is never very extensive and in standard conditions few c-Fos positive cells are detected. Indeed in our experimental conditions, no significant recruitment could be induced by monocular ON-OFF light stimulation, suggesting that either the intrinsic properties of dLGN cells or a mixture of excitation and inhibition at the level of the retina and/or dLGN could prevent a sufficient firing in thalamocortical cells or in neighboring interneurons. On the other hand, this might be simply due to the low activity resolution of c-Fos expression measurement, which can only highlight intense activity of neural cells. However, to our knowledge, besides the very recently developed Campari method (Fosque et al., 2015), which would have required rat transgenesis, c-Fos is still an extremely useful method when it comes to characterizing the spatial position and the phenotype of such large networks of active/inactive cells

To highlight this regulatory properties of dLGN we wanted to eliminate the upstream filtering of the retinal circuitry. From *in vitro* experiments on isolated retinas we found that 4-AP, an A-type potassium channel blocker, is able to increase the activity of RGCs in a non-regulated manner, leading to a parallel independent activation of the two ON-OFF retinal channels and effectively shunting retinal cross regulation. When 4-AP was administered *in vivo* via intravitreal injection the number of active cells found in the dLGN was strongly increased. As a confirmation of the chaotic and non-stimulus dependent activation of RGCs exposed to 4-AP, no significant differences could be found between the no-light and light stimulated animals. Also, no stimulus dependent activity could be detected via VEP recordings. One interesting aspect emerged from the analysis of 4-AP experiments. With or without light stimulation roughly 70% of c-Fos positive cells were found to be NeuN negative. The use of antibodies against the GABA synthesizing enzymes (GAD65 and GAD 67), a reliable markers of GABAergic cells (Meyer et al., 2011), provided clear evidence that many active cells were GABAergic interneurons, and that most of these GABAergic cells (53%) were NeuN negative. Previous reports have provided clear evidence that GABAergic interneurons are functionally heterogeneous (Williams et al., 1996). An opposite behavior emerged when RGCs where exposed to L-AP4, an mGlur6 receptor agonist which is known to block the ON retinal pathway (Slaughter and Miller, 1981). In this latter case the ON component of VEP recordings was abolished while leaving the OFF component almost unaltered. Also, a clear difference between the no light and light condition could be detected, with a much weaker recruitment of GABAergic interneurons than with 4-AP and a diffused stimulus driven activation of NeuN positive neurons within the dLGN when compared to the no-light condition. These results suggest the existence of a population of GABAergic interneurons belonging to a strong inhibitory circuitry that is set to filter out the visual signal in the presence of non-coherent tonic RGC firing, as in the case of 4-AP exposure. On the contrary, a single channel (OFF) input to dLGN, as in the case of L-AP4 administration upon light stimulation, might be less effective in recruiting such inhibitory circuitry, leading to a stimulus induced activation of dLGN neurons that is even stronger than in the no drug condition. However, it has to be noticed that an additional explanation to such stronger recruitment of dLGN cell could also arise from the suppression of channel cross inhibition within the retina itself when exposed to L-AP4 (Margolis and Detwiler, 2007; Liang and Freed, 2010). In the future it would be important to clarify the contribution of all these components to this functional behavior, possibly attributing specific roles to one or more of the previously described categories of GABAergic interneurons of the dLGN (Williams et al., 1996). Also it would be important to ascertain if thalamocortical cells participate in the filtration process of tonic and non-coherent visual inputs, by their transition from bursting firing mode to a less effective tonic discharge, because of tonic depolarization with inactivation of *T*-type voltage activated calcium channels and/or deactivation of Hyperpolarization-activated cyclic nucleotide-gated (HCN) channels (Jahnsen and Llinás, 1984; McCormick and Feeser, 1990; McCormick and Pape, 1990). At this stage, considering the complexity of the thalamic circuitry, the different variety of GABAergic cells (Williams et al., 1996), the many modulatory pathways and the short- and long-feedback loops impinging upon thalamocortical neurons (Burke and Jervie Sefton, 1966; Berardi and Morrone, 1984; Murphy et al., 1999; Blitz and Regehr, 2003, 2005; Chen and Regehr, 2003; Cudeiro and Sillito, 2006; Hammer et al., 2014), it is difficult to provide a conclusive explanation without further experimental evidence. Our results apparently contrast with previous findings (Montero and Jian, 1995) that showed no significant difference in the soma areas of c-Fos positive and c-Fos negative cells in the dLGN after light stimulation. In that case it was concluded that small interneurons and larger relay cells were equally activated. It has to be noted, however, that these results are not directly comparable: Montero and Jian stimulated using a campimeter and were able to detect a large number of c-Fos positive cells in the pigmented rat and did not use any drug. Preferential activation of cell subtypes, as in our case, might be highlighted with c-Fos expression only after pharmacological manipulation aimed at pushing this neural system to its physiological limits, eventually recruiting its regulatory circuitry to a large extent.

Regarding dLGN neurons, which were identified as NeuN positive, presumably thalamocortical neurons, our anatomical staining suggests that no spatial clustering and/or segregation of these cells are detectable. When this analysis was extended to functional segregation, we found evidence for non-homogeneous distribution of active cells inside the dLGN structure in the 4-AP and L-AP4 experiments (c-Fos/NeuN double positive cells). In most cases a significant lateralization of active neurons, with a higher density of active cells near the lateral border of the dLGN, was detected. It is not easy to explain how such functional segregation could be generated and what its role might be in the processing of the visual information, although some functional segregation of visual features processing has been reported in the dLGN (Dhande and Huberman, 2014; Bickford et al., 2015). This external functional lamina seems to correlate with the entry point of retino-thalamic axons known to access the dLGN from the lateral edge. Despite these considerations, our results do not correlate with the presence of contralateral axons also on the medial border of the dLGN (Reese, 1988; Kim et al., 2010). This functional segregation of active neurons in the dLGN might therefore reflect a possible gradient of inhibition which gets stronger in the lateral-medial direction, possibly due to either the intrinsic inhibitory circuitry or to the cortical-thalamic inhibitory feedback, whose fibers are known to enter the dLGN from its medial border (Su et al., 2011).

## Author contributions

AAr, MB, GM, MR, MF performed and design the experiments; AAm, JL, GM performed data analysis; AM designed research; AM, GM, and VZ wrote the paper.

### Conflict of interest statement

The authors declare that the research was conducted in the absence of any commercial or financial relationships that could be construed as a potential conflict of interest.

## Supplementary material

Supplementary Figure 1 **Exemplar light induced ON and OFF responses in a rat ON-OFF RGC**. Spike recordings and the effect of light pulses (1 s) in two exemplar ON-OFF RGCs. Top lines are the schematic representation of the light stimulus. The middle and bottom track are representative spike recording before (middle) and during (bottom) the application of L-AP4 (20 μM). Notice how the application of L-AP4 abolishes the ON response leaving the OFF response unaltered. Black scale bar is equal to 1 s.

Supplementary Figure 2 **Distribution of c-Fos Fluorescence-Threshold ratio for GAD positive cells**. Histograms representing the distribution of c-Fos fluorescence intensity of GAD positive cells divided by the field specific threshold used for selection of highly active cells [Threshold = Mean (Background) + 2 x std(Background)]. The red vertical lines divide the highly active from the lowly active GAD positive cells. Highly active cells are represented with red bars. On the y axis normalized frequencies are reported. The top histogram refers to cells from L-AP4 injected animals, while bottom histogram refers to cells from 4-AP injected animals. The number of GAD positive cells is reported in each histogram. Notice how the percentage of highly active cells is greatly increased in the 4-AP group.

Supplementary Figure 3 **LGN neuronal activity pattern in control conditions (A,B)**. Representative c-Fos immunostainings of the right LGN (R) and digital reconstructions from the same coronal sections to visualize active neurons in the right (R) and left (L) LGNs from rats kept in darkness **(A)** or light-stimulated **(B)** with alternating black and white vertical bars at constant overall luminance (white bars 37 mW/m^2^; black bars 0.11 mW/m^2^; 2 h; 2 Hz refresh rate; 0.5 cycle/degree; left eye stimulation). The three small panels on the right are magnification of the dLGN, IGL, and vLGN from the corresponding sections. Continuous and dashed lines indicate edges of dLGN, IGL, and vLGN. In the digital reconstruction, circles report the location of identified c-Fos positive cells (for segmentation algorithm, see Materials and Methods). Few spare cells are active in the dLGN in the no-light and following ON-OFF light-stimulation, while clear activity is detected in the IGL and vLGN. The calibration bar is 200 μm for the large immunostaining panels and 50 μm for the small insets.

Supplementary Figure 4 **NeuN, GAD, and c-Fos staining for the reticular nucleus**. Since the reticular nucleus is one of the main inhibitory input to the dLGN, this nucleus was inspected to assess if the lack of c-Fos expression following visual stimulation in the dLGN could be due to its strong activation. This is an exemplar image from a rat after monocular visual stimulation (see Materials and Methods). **(A)** Double staining for GAD (on the left) and c-Fos (on the right), clearly showing the lack of c-Fos expression by GAD positive cells. **(B)** Double staining for GAD (on the left) and NeuN (on the right), clearly showing how, in the reticular nucleus, differently from the dLGN, all GAD+ cells are intensely stained by NeuN.
