# Supplementary figures and images for: Effects of the Concomitant Activation of ON and OFF Retinal Ganglion Cells on the Visual Thalamus: Evidence for an Enhanced Recruitment of GABAergic Cells
Source: Front Neural Circuits. 2015 Nov 24;9:77. doi: 10.3389/fncir.2015.00077 (PMC4656840; doi:10.3389/fncir.2015.00077)

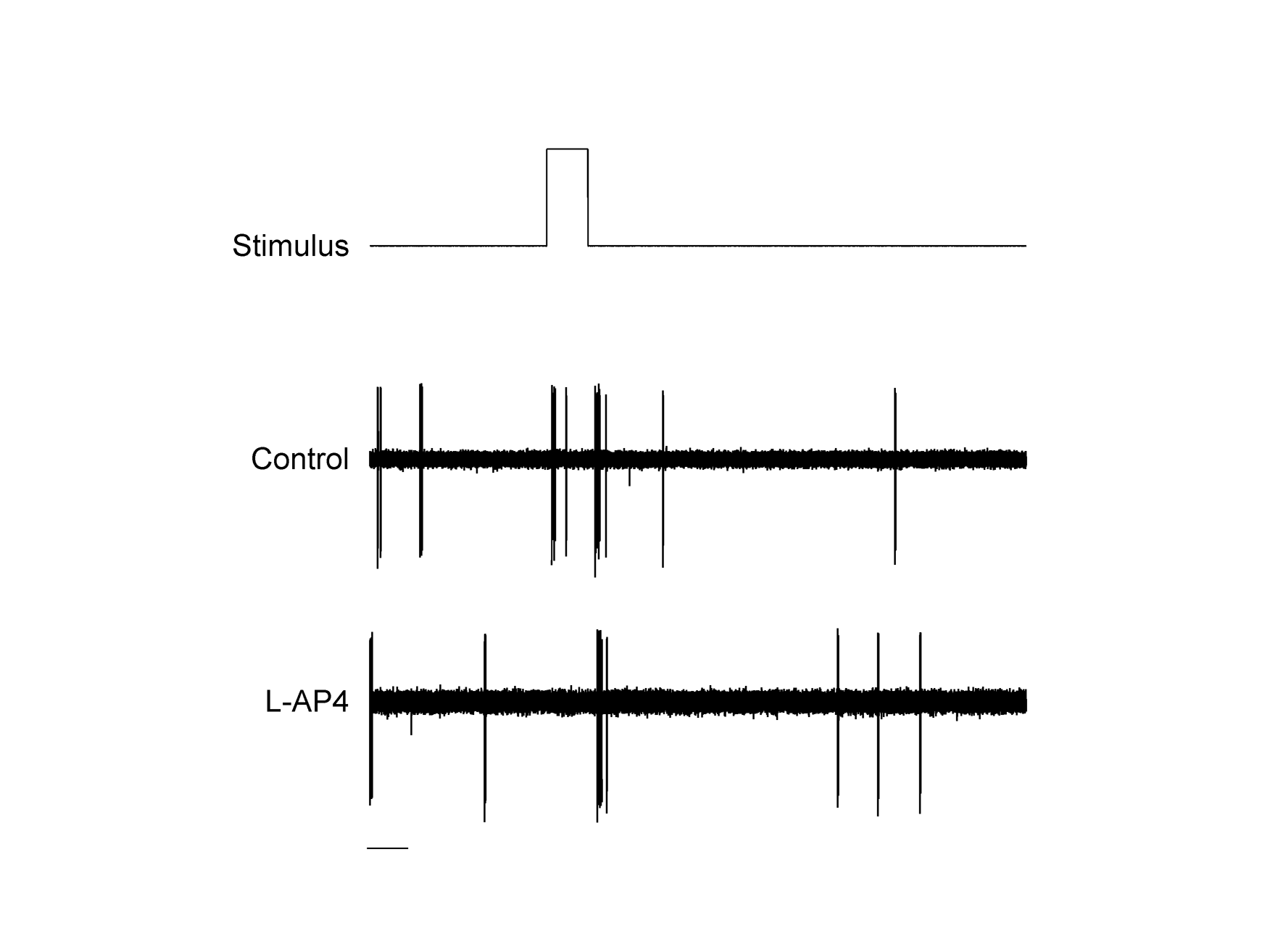

Supplement: Supplementary Figure 1 — Exemplar light induced ON and OFF responses in a rat ON-OFF RGC. Spike recordings and the effect of light pulses (1 s) in two exemplar ON-OFF RGCs. Top lines are the schematic representation of the light stimulus. The middle and bottom track are representative spike recording before (middle) and during (bottom) the application of L-AP4 (20 μM). Notice how the application of L-AP4 abolishes the ON response leaving the OFF response unaltered. Black scale bar is equal to 1 s. [file Image1.TIF]

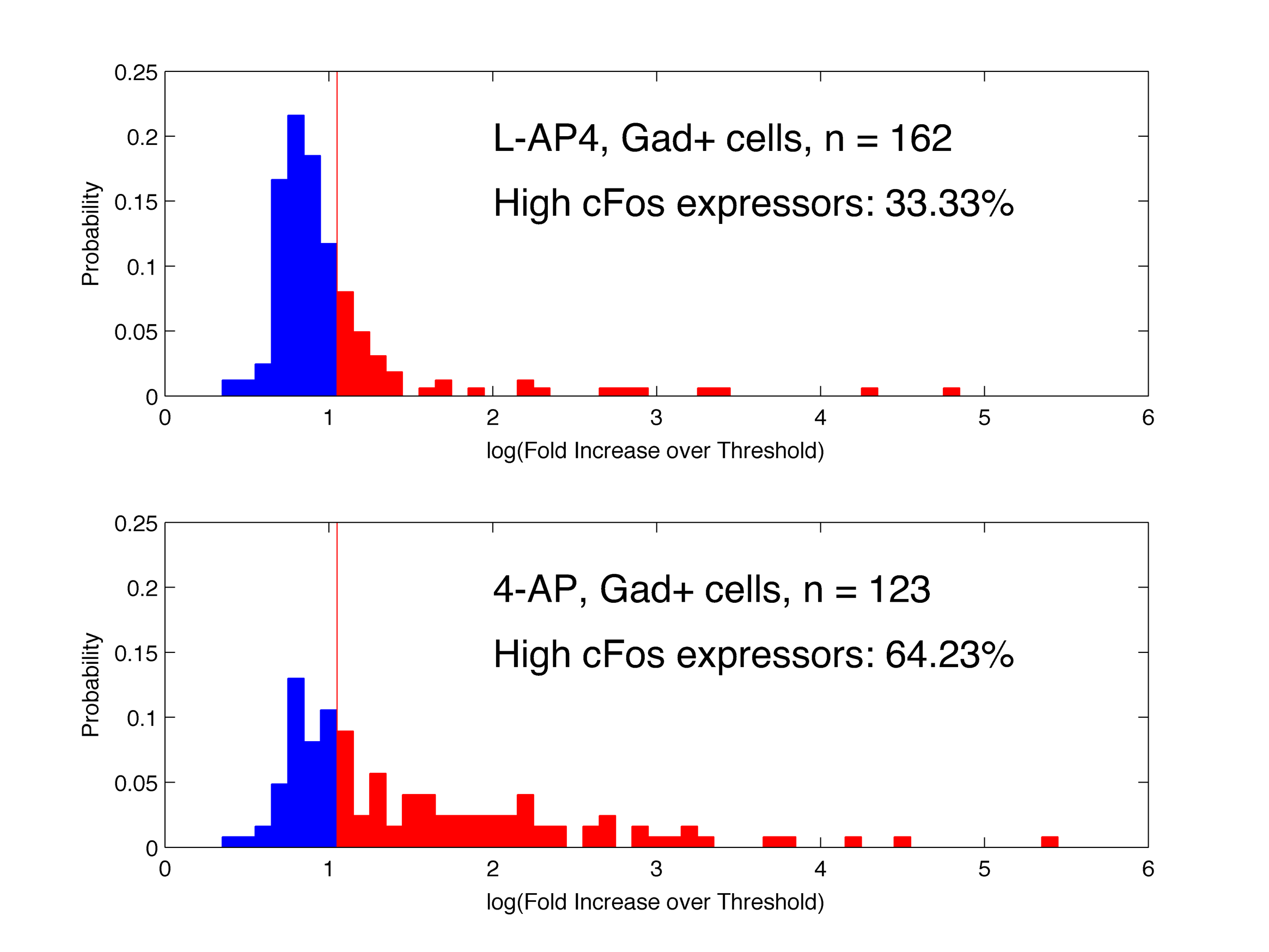

Supplement: Supplementary Figure 2 — Distribution of c-Fos Fluorescence-Threshold ratio for GAD positive cells. Histograms representing the distribution of c-Fos fluorescence intensity of GAD positive cells divided by the field specific threshold used for selection of highly active cells [Threshold = Mean (Background) + 2 x std(Background)]. The red vertical lines divide the highly active from the lowly active GAD positive cells. Highly active cells are represented with red bars. On the y axis normalized frequencies are reported. The top histogram refers to cells from L-AP4 injected animals, while bottom histogram refers to cells from 4-AP injected animals. The number of GAD positive cells is reported in each histogram. Notice how the percentage of highly active cells is greatly increased in the 4-AP group. [file Image2.TIF]

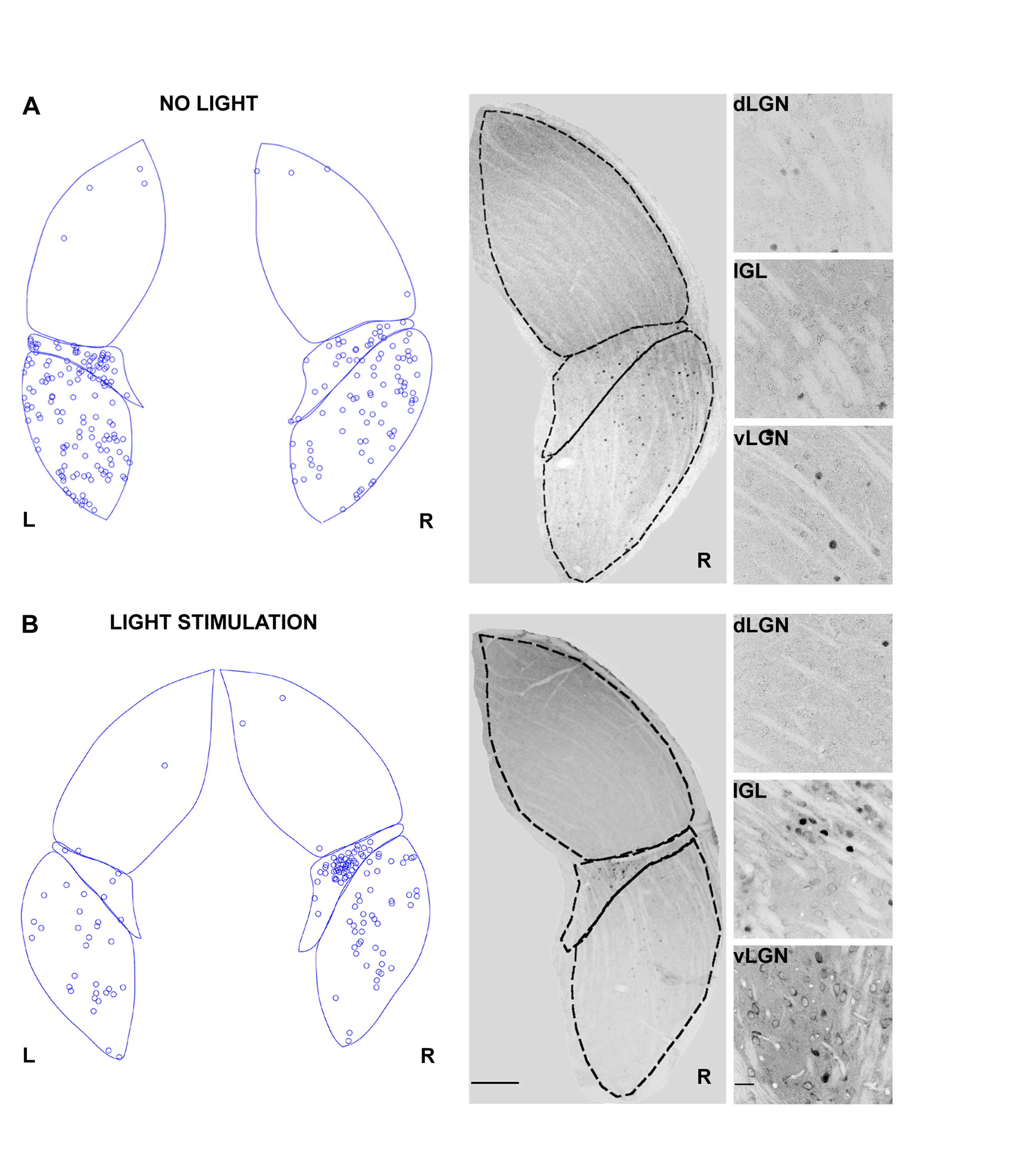

Supplement: Supplementary Figure 3 — LGN neuronal activity pattern in control conditions (A,B). Representative c-Fos immunostainings of the right LGN (R) and digital reconstructions from the same coronal sections to visualize active neurons in the right (R) and left (L) LGNs from rats kept in darkness (A) or light-stimulated (B) with alternating black and white vertical bars at constant overall luminance (white bars 37 mW/m2; black bars 0.11 mW/m2; 2 h; 2 Hz refresh rate; 0.5 cycle/degree; left eye stimulation). The three small panels on the right are magnification of the dLGN, IGL, and vLGN from the corresponding sections. Continuous and dashed lines indicate edges of dLGN, IGL, and vLGN. In the digital reconstruction, circles report the location of identified c-Fos positive cells (for segmentation algorithm, see Materials and Methods). Few spare cells are active in the dLGN in the no-light and following ON-OFF light-stimulation, while clear activity is detected in the IGL and vLGN. The calibration bar is 200 μm for the large immunostaining panels and 50 μm for the small insets. [file Image3.TIF]

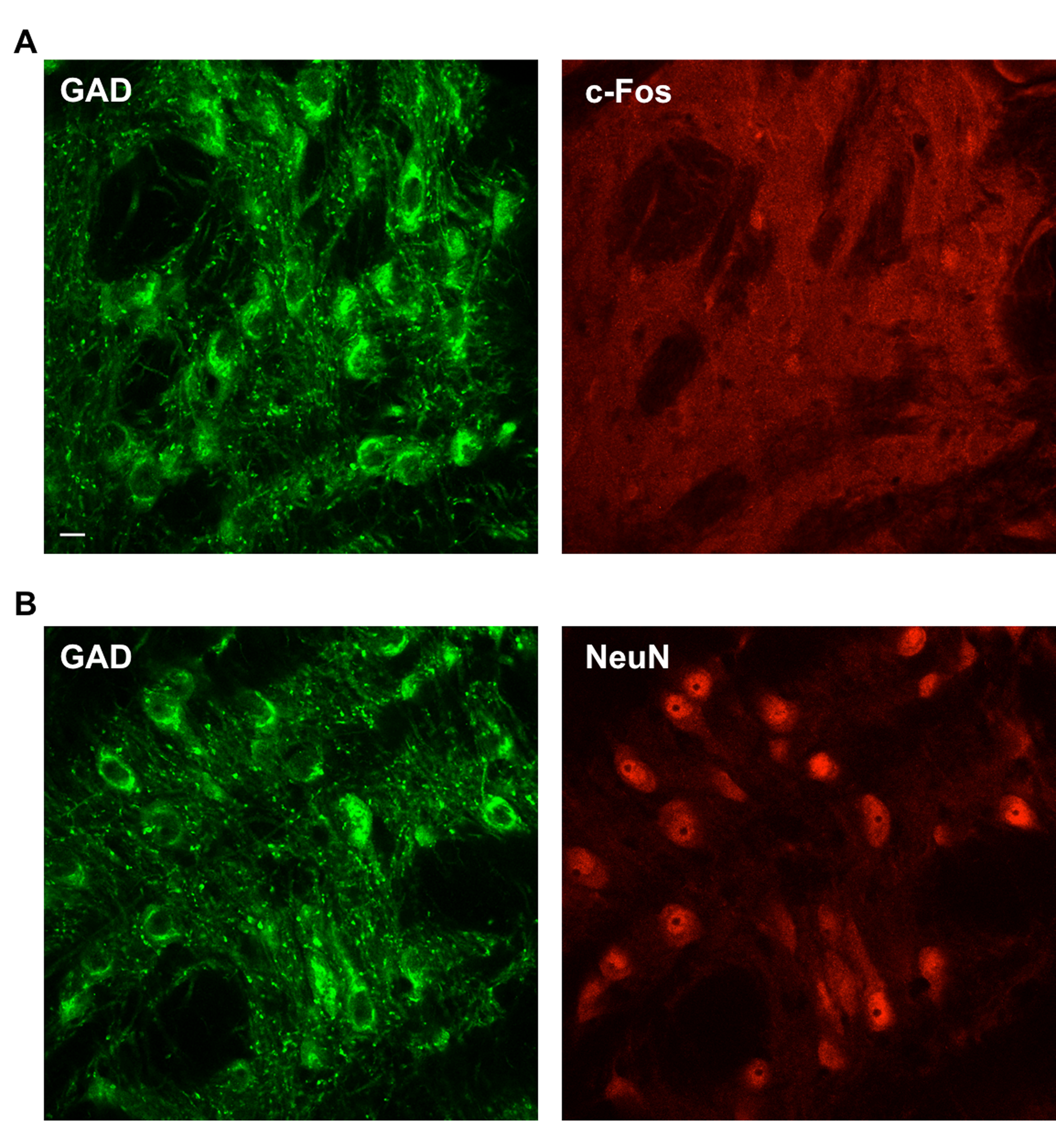

Supplement: Supplementary Figure 4 — NeuN, GAD, and c-Fos staining for the reticular nucleus. Since the reticular nucleus is one of the main inhibitory input to the dLGN, this nucleus was inspected to assess if the lack of c-Fos expression following visual stimulation in the dLGN could be due to its strong activation. This is an exemplar image from a rat after monocular visual stimulation (see Materials and Methods). (A) Double staining for GAD (on the left) and c-Fos (on the right), clearly showing the lack of c-Fos expression by GAD positive cells. (B) Double staining for GAD (on the left) and NeuN (on the right), clearly showing how, in the reticular nucleus, differently from the dLGN, all GAD+ cells are intensely stained by NeuN. [file Image4.TIF]
